# Investigation of Genetic Relationships Between *Hanseniaspora* Species Found in Grape Musts Revealed Interspecific Hybrids With Dynamic Genome Structures

**DOI:** 10.3389/fmicb.2019.02960

**Published:** 2020-01-15

**Authors:** Méline Saubin, Hugo Devillers, Lucas Proust, Cathy Brier, Cécile Grondin, Martine Pradal, Jean-Luc Legras, Cécile Neuvéglise

**Affiliations:** ^1^Micalis Institute, INRA, AgroParisTech, Université Paris-Saclay, Jouy-en-Josas, France; ^2^Micalis Institute, INRA, AgroParisTech, CIRM-Levures, Université Paris-Saclay, Jouy-en-Josas, France; ^3^SPO, Univ Montpellier, INRA, Montpellier SupAgro, Montpellier, France

**Keywords:** MLST, yeast, biodiversity, evolution, *Hanseniaspora uvarum*, *Hanseniaspora guilliermondii*

## Abstract

*Hanseniaspora*, a predominant yeast genus of grape musts, includes sister species recently reported as fast evolving. The aim of this study was to investigate the genetic relationships between the four most closely related species, at the population level. A multi-locus sequence typing strategy based on five markers was applied on 107 strains, confirming the clear delineation of species *H. uvarum, H. opuntiae, H. guilliermondii*, and *H. pseudoguilliermondii*. Huge variations were observed in the level of intraspecific nucleotide diversity, and differences in heterozygosity between species indicate different life styles. No clear population structure was detected based on geographical or substrate origins. Instead, *H. guilliermondii* strains clustered into two distinct groups, which may reflect a recent step toward speciation. Interspecific hybrids were detected between *H. opuntiae* and *H. pseudoguilliermondii*. Their characterization using flow cytometry, karyotypes and genome sequencing showed different genome structures in different ploidy contexts: allodiploids, allotriploids, and allotetraploids. Subculturing of an allotriploid strain revealed chromosome loss equivalent to one chromosome set, followed by an auto-diploidization event, whereas another auto-diploidized tetraploid showed a segmental duplication. Altogether, these results suggest that *Hanseniaspora* genomes are not only fast evolving but also highly dynamic.

## Introduction

Grape must is a complex ecosystem that combines grape and cellar micro-organisms. Many species are interacting with each other, including yeasts and bacteria (Jolly et al., 2014; Capozzi et al., 2015; Raymond Eder et al., 2017). It is now well recognized that natural microbial populations play an important role in winemaking, notably by increasing the complexity of wine aromas (Gamero et al., 2016; Padilla et al., 2016; Bagheri et al., 2018; Escribano et al., 2018). The occurrence of microorganisms in grapes is dependent on biotic and abiotic factors such as geographic location, soil, grapevine cultivar, viticultural practices, and climate (Bokulich et al., 2014; Drumonde-Neves et al., 2016). Yeast biodiversity in must is thus generally vintage dependent (Vigentini et al., 2015). Whereas *Saccharomyces cerevisiae*, already well known for its importance in diverse fermented food production, is the dominant species at the end of vinification, the yeast species commonly found on grapes and in musts at the beginning of spontaneous fermentations are rather Saccharomycotina than Basidiomycotina. They mostly belong to genera *Hanseniaspora, Lachancea, Metschnikowia, Pichia*, and *Starmerella* (Wang et al., 2015; Varela and Borneman, 2017; Cioch-Skoneczny et al., 2018; Lorenzini and Zapparoli, 2019), and the genus *Hanseniaspora* is generally the most abundant at the onset of the fermentation.

The Saccharomycotina genus *Hanseniaspora* belongs to the Saccharomycodaceae family. Due to their lemon-shaped cell structure, *Hanseniaspora* species have been called apiculate yeasts, together with the closely related species of the genera *Saccharomycodes* and *Nadsonia.* The genus includes 21 described species, which can be separated into two lineages based on phylogenetic relationships deduced from D1D2 domains of the 28S rRNA subunit (Boekhout et al., 1994; Ouoba et al., 2015; Martin et al., 2018) or from the concatenation of taxonomic markers (Cadez et al., 2019). Recently, based on whole genome sequence comparison, Steenwyk et al. (2019) confirmed *Hanseniaspora* to be composed of two lineages, a fast-evolving lineage (FEL) and a slow-evolving lineage (SEL), which differ by their evolution rate and the extent of their gene loss. Species found on grapes and musts belong to both FEL (mostly *H. uvarum*) and SEL (*H. vinae*). Several studies report that they play an important role in wine fermentation by producing flavors, modulating the growth and metabolism of *Saccharomyces cerevisiae* and affecting wine color (reviewed in Martin et al., 2018). However, population structure and genetic relationships between *Hanseniaspora* species remain unclear. Nine species of FEL are particularly close phylogenetically and some of them could be difficult to differentiate probably because they diverged quite recently (Cadez et al., 2002). D1D2 regions of the ribosomal subunit, which are generally used for taxonomic classification and phylogenetic trees, differ by less than seven nucleotides between pairs of species and only by two nucleotides between *H. opuntiae* and *H. guilliermondii*, and between *H. meyeri* and *H. clermontiae*.

Four of these closely related species are associated with grapes and wine environment: *H. uvarum, H. opuntiae, H. pseudoguilliermondii*, and *H. guilliermondii*. Although frequently found in grapes, population studies have been performed only in *H. uvarum* (Albertin et al., 2016). To clarify the phylogenetic relationships within this species complex, and eventually to detect hidden sub-species clustering, we first designed multi locus sequence typing (MLST) markers to distinguish strains at the inter- and intraspecies level. MLST method allows comparison of variable sequences between strains or species inside highly conserved housekeeping genes. This method, initially developed for the identification of clones within populations of pathogenic bacteria (Maiden et al., 1998), is progressively becoming classic for genomic diversity studies in yeasts from different environments, and many markers have been reported (Bougnoux et al., 2002; Ayoub et al., 2006; Odds and Jacobsen, 2008; Jacques et al., 2017; Tittarelli et al., 2018). In this study, MLST analysis performed on *Hanseniaspora* strains was used to clarify the complex of the four wine-growing species examined and allowed detection of inter-specific hybrids, which were then investigated by flow cytometry and karyotyping. Finally, genome sequencing and comparison to parental species genomes provided more details into the contribution of each of them to the genome of the hybrids.

## Materials and Methods

### *Hanseniaspora* Strains

A total of 120 *Hanseniaspora* strains were examined in this study, 35 of them were collected from French grape must in Occitanie region, France in 2015 and 2016 (Table 1). The other strains have been collected from grape musts or from other substrates in distant French regions and in countries mainly from Europe, South America and Africa. Yeast cells were cultivated on YPD medium (yeast extract 10 g/L, peptone 10 g/L, glucose 10 g/L) at 28°C.

**TABLE 1:** List of strains.

**Genus**	**Species**	**Name**	**Synonyms**	**Substrate**	**Country**	**Isolation date**
*Hanseniaspora*	*guilliermondii*	11-1173		Toumodi Vin de rônier	Ivory Coast	2016
*Hanseniaspora*	*guilliermondii*	11-1176		Flower	Ivory Coast	2016
*Hanseniaspora*	*guilliermondii*	CBS 1972		Grape juice	Italy	<1978^∗^
*Hanseniaspora*	*guilliermondii*	CBS 2591		Trachea of bee	France	<1978^∗^
*Hanseniaspora*	*guilliermondii*	CBS 95		Fermenting bottled tomatoes	Netherlands	<1978^∗^
*Hanseniaspora*	*guilliermondii*	CLIB 510^T^	CBS 465^T^	Infected nail	South Africa	1978
*Hanseniaspora*	*guilliermondii*	CLIB 1559		Lemon	French Guiana	2010
*Hanseniaspora*	*guilliermondii*	CLIB 3085		Toumodi Vin de rônier	Ivory Coast	2016
*Hanseniaspora*	*guilliermondii*	CLIB 3092	V2-10	Grape must Viognier	France (Sommières, Occitanie)	2015
*Hanseniaspora*	*guilliermondii*	CLIB 3093	S1-2	Grape must Sauvignon	France (Puylacher, Occitanie)	2015
*Hanseniaspora*	*guilliermondii*	CLIB 3094	S1-3	Grape must Sauvignon	France (Puylacher, Occitanie)	2015
*Hanseniaspora*	*guilliermondii*	CLIB 3095	S2-34	Grape must Sauvignon	France (Saint Mathieu de Tréviers, Occitanie)	2015
*Hanseniaspora*	*guilliermondii*	CLIB 3096	S4-9	Grape must Sauvignon	France (Ouveillan, Occitanie)	2015
*Hanseniaspora*	*guilliermondii*	CLIB 3097	S4-16	Grape must Sauvignon	France (Ouveillan, Occitanie)	2015
*Hanseniaspora*	*guilliermondii*	CLIB 3098	V2-1	Grape must Viognier	France (Sommières, Occitanie)	2015
*Hanseniaspora*	*guilliermondii*	CLIB 3099	V2-2	Grape must Viognier	France (Sommières, Occitanie)	2015
*Hanseniaspora*	*guilliermondii*	CLIB 3100	V5-12	Grape must Viognier	France (Pech Rouge, Occitanie)	2015
*Hanseniaspora*	*guilliermondii*	CLIB 3206	V1-9	Grape must Viognier	France (Saint Mathieu de Tréviers, Occitanie)	2015
*Hanseniaspora*	*guilliermondii*	CLIB 3207	BIA-V1L15	Grape must Viognier	France (Saint Mathieu de Tréviers, Occitanie)	2015
*Hanseniaspora*	*guilliermondii*	CLIB 3208	V5-13	Grape must Viognier	France (Pech Rouge, Occitanie)	2015
*Hanseniaspora*	*guilliermondii*	CLIB 3209	V5-30	Grape must Viognier	France (Pech Rouge, Occitanie)	2015
*Hanseniaspora*	*guilliermondii*	CLIB 3210	S4-54	Grape must Sauvignon	France (Ouveillan, Occitanie)	2015
*Hanseniaspora*	*guilliermondii*	CLIB 3228	R18-212	Fermented juice of sugar cane (3 days)	Réunion Island	2018
*Hanseniaspora*	*opuntiae x pseudoguilliermondii*	CCY46-1-3		Fruit; plum tree (Prunus domestica L. ‘Stanley’)	Slovakia (Malé Leváre)	2009
*Hanseniaspora*	*opuntiae x pseudoguilliermondii*	CCY46-1-3a		Single cell colony of CCY46-1-3	Lab strain	2019
*Hanseniaspora*	*opuntiae x pseudoguilliermondii*	CCY46-1-3b		Single cell colony of CCY46-1-3	Lab strain	2019
*Hanseniaspora*	*opuntiae x pseudoguilliermondii*	CCY46-1-3c		Single cell colony of CCY46-1-3	Lab strain	2019
*Hanseniaspora*	*opuntiae x pseudoguilliermondii*	CCY46-1-3d		Single cell colony of CCY46-1-3	Lab strain	2019
*Hanseniaspora*	*opuntiae x pseudoguilliermondii*	CLIB 3101	V3-28	Grape must Viognier	France (Puylacher, Occitanie)	2015
*Hanseniaspora*	*opuntiae x pseudoguilliermondii*	DBVPG 5828		Soil close to plum tree	Algeria	2010
*Hanseniaspora*	*opuntiae x pseudoguilliermondii*	CLIB 3263	M18-204	Fermented pineapple	Mayotte Island	2018
*Hanseniaspora*	*opuntiae x pseudoguilliermondii*	CLIB 3313	M18-207	Fermented pineapple	Mayotte Island	2018
*Hanseniaspora*	*opuntiae x pseudoguilliermondii*	CLIB 3265	M18-215	Fermented pineapple	Mayotte Island	2018
*Hanseniaspora*	*opuntiae*	11-1102		Fruit papaya	Guatemala (Flores)	2009
*Hanseniaspora*	*opuntiae*	11-1139		Fallen fruit	El Salvador (San Salvador)	2009
*Hanseniaspora*	*opuntiae*	11-1184		Flower (Hybiscus)	Palau (Ngerekebesang)	2010
*Hanseniaspora*	*opuntiae*	11-1196		Rotten fruit (*Syzygium malaccense*)	Palau (Koror)	2010
*Hanseniaspora*	*opuntiae*	CLIB 1203		Ivy	French Guiana	2008
*Hanseniaspora*	*opuntiae*	CLIB 1208		Papaya	French Guiana	2008
*Hanseniaspora*	*opuntiae*	CLIB 1557		Papaya	French Guiana	2010
*Hanseniaspora*	*opuntiae*	MUCL 49139^T^	CBS 8733^T^	Rot	Hawaii Island	<2003^∗^
*Hanseniaspora*	*opuntiae*	CLIB 1564		Ant	French Guiana	2010
*Hanseniaspora*	*opuntiae*	CLIB 3102	V1-109	Grape must Viognier	France (Saint Mathieu de Tréviers, Occitanie)	2015
*Hanseniaspora*	*opuntiae*	CLIB 3103	V3-16	Grape must Viognier	France (Puylacher, Occitanie)	2015
*Hanseniaspora*	*opuntiae*	CLIB 3104	V4-31	Grape must Viognier	France (Fabrezan, Occitanie)	2015
*Hanseniaspora*	*opuntiae*	CLIB 3105	S1-11	Grape must Sauvignon	France (Puylacher, Occitanie)	2015
*Hanseniaspora*	*opuntiae*	CLIB 3106	S1-14	Grape must Sauvignon	France (Puylacher, Occitanie)	2015
*Hanseniaspora*	*opuntiae*	CLIB 3107	BIA-S2L2	Grape must Sauvignon	France (Saint Mathieu de Tréviers, Occitanie)	2015
*Hanseniaspora*	*opuntiae*	CLIB 3108	V1-1	Grape must Viognier	France (Saint Mathieu de Tréviers, Occitanie)	2015
*Hanseniaspora*	*opuntiae*	CLIB 3109	V3-27	Grape must Viognier	France (Puylacher, Occitanie)	2015
*Hanseniaspora*	*opuntiae*	CLIB 3203	V2-12	Grape must Viognier	France (Sommières, Occitanie)	2015
*Hanseniaspora*	*opuntiae*	CLIB 3204	V2-31	Grape must Viognier	France (Sommières, Occitanie)	2015
*Hanseniaspora*	*opuntiae*	CLIB 3205	S2-25	Grape must Sauvignon	France (Saint Mathieu de Tréviers, Occitanie)	2015
*Hanseniaspora*	*opuntiae*	CLIB 3227	R18-143	”Grenadille” fruit	Réunion Island	2018
*Hanseniaspora*	*opuntiae*	CLIB 3230	R18-219	Lemonade (3 days fermentation)	Réunion Island	2018
*Hanseniaspora*	*opuntiae*	CLIB 3234	R18-484	Lemonade (3 days fermentation)	Réunion Island	2018
*Hanseniaspora*	*pseudoguilliermondii*	11-494		Fruit papaya	The Philippines (Manila)	2011
*Hanseniaspora*	*pseudoguilliermondii*	CBS 8772^T^		Orange juice concentrate	unknown	<2006^∗^
*Hanseniaspora*	*pseudoguilliermondii*	CLIB 1441		Orange	French Guiana	2010
*Hanseniaspora*	*pseudoguilliermondii*	CLIB 3226	R18-113	Fruit Evi	Réunion Island	2018
*Hanseniaspora*	*pseudoguilliermondii*	CLIB 3229	R18-218	Lemonade (3 days fermentation)	Réunion Island	2018
*Hanseniaspora*	*pseudoguilliermondii*	CLIB 3233		Fruit	French Guiana	2010
*Hanseniaspora*	*uvarum*	10-1471		Grape	Slovakia (Malá Tr̀ňa)	2015
*Hanseniaspora*	*uvarum*	11-1148		Flower	Guatemala (Guatemala City)	2009
*Hanseniaspora*	*uvarum*	11-1288		Flower (Leguminosae)	Romania (Bucuresti)	2006
*Hanseniaspora*	*uvarum*	A1		Grape must Pinot noir Côte de nuit	France (Burgundy)	2005
*Hanseniaspora*	*uvarum*	A4		Grape must Pinot noir Côte de nuit	France (Burgundy)	2005
*Hanseniaspora*	*uvarum*	B2		Grape must Pinot noir Côte de nuit	France (Burgundy)	2005
*Hanseniaspora*	*uvarum*	C4		Grape must Pinot noir Côte de beaune	France (Burgundy)	2005
*Hanseniaspora*	*uvarum*	CBS 2583		Fermenting cucumber brine	United States	?
*Hanseniaspora*	*uvarum*	CBS 2585		Sour dough	Portugal	1978
*Hanseniaspora*	*uvarum*	CBS 2588		Tanning fluid	France	?
*Hanseniaspora*	*uvarum*	CBS 286		Soil	Indonesia	<1934^∗^
*Hanseniaspora*	*uvarum*	CCY25-6-34		Grape must (7 days fermentation), variety Green Veltliner	Slovakia (Strekov)	2008
*Hanseniaspora*	*uvarum*	CCY25-6-36		Soil adjacent to apricot tree	Slovakia (Malé Zálužie)	2013
*Hanseniaspora*	*uvarum*	CCY46-1-2		Fresh-water lake	Slovakia (Plaveckẏ Štvrtok)	1987
*Hanseniaspora*	*uvarum*	CLIB 303^T^	CBS 314^T^	Muscatel grape	Ukraine	<1978^∗^
*Hanseniaspora*	*uvarum*	CLIB 512		Soil	Danemark	1978
*Hanseniaspora*	*uvarum*	CLIB 979		Sylvaner start AF	France (Alsace)	2001
*Hanseniaspora*	*uvarum*	CLIB 1207		Cacao Berry	French Guiana	2008
*Hanseniaspora*	*uvarum*	CLIB 1209		Saul flower	French Guiana	2008
*Hanseniaspora*	*uvarum*	CLIB 1210		Saul flower	French Guiana	2008
*Hanseniaspora*	*uvarum*	CLIB 1563		Butterfly	French Guiana	2010
*Hanseniaspora*	*uvarum*	CLIB 1627		Saul flower	French Guiana	2008
*Hanseniaspora*	*uvarum*	CLIB 1650		Vineyard	France (Alsace)	2007
*Hanseniaspora*	*uvarum*	CLIB 3110	S1-8	Grape must Sauvignon	France (Puylacher, Occitanie)	2015
*Hanseniaspora*	*uvarum*	CLIB 3111	V1-6	Grape must Viognier	France (Saint Mathieu de Tréviers, Occitanie)	2015
*Hanseniaspora*	*uvarum*	CLIB 3114	V7-44	Grape must Viognier	France (Pech Rouge, Occitanie)	2016
*Hanseniaspora*	*uvarum*	CLIB 3115	S6-45	Grape must Sauvignon	France (Pech Rouge, Occitanie)	2016
*Hanseniaspora*	*uvarum*	CLIB 3117	V4-17	Grape must Viognier	France (Fabrezan, Occitanie)	2015
*Hanseniaspora*	*uvarum*	CLIB 3118	V3-8	Grape must Viognier	France (Puylacher, Occitanie)	2015
*Hanseniaspora*	*uvarum*	CLIB 3119	dV7-11	Grape must Viognier	France (Pech Rouge, Occitanie)	2016
*Hanseniaspora*	*uvarum*	CLIB 3120	dV7-39	Grape must Viognier	France (Pech Rouge, Occitanie)	2016
*Hanseniaspora*	*uvarum*	CLIB 3123	dV7-66	Grape must Viognier	France (Pech Rouge, Occitanie)	2016
*Hanseniaspora*	*uvarum*	CLIB 3224	R18-8	Coffee bean	Réunion Island	2018
*Hanseniaspora*	*uvarum*	CLIB 3225	R18-18	Fermented coffee	Réunion Island	2018
*Hanseniaspora*	*uvarum*	CLIB 3231	R18-221	Grape	Réunion Island	2018
*Hanseniaspora*	*uvarum*	CLIB 3232	R18-409	Badamier fruit	Réunion Island	2018
*Hanseniaspora*	*uvarum*	CLIB 3237	R18-2	Coffee bean	Réunion Island	2018
*Hanseniaspora*	*uvarum*	CLIB 3239	R18-388	Grape	Réunion Island	2018
*Hanseniaspora*	*uvarum*	CLIB 3241	R18-185	Badamier fruit	Réunion Island	2018
*Hanseniaspora*	*uvarum*	D8		Grape must Pinot noir Côte de beaune	France (Burgundy)	2005
*Hanseniaspora*	*uvarum*	E6		Grape must Pinot noir Côte de Chalonnais	France (Burgundy)	2005
*Hanseniaspora*	*uvarum*	MAFF 516149		Unknown	Japan	<2016^∗^
*Hanseniaspora*	*uvarum*	NZ15		Grape must	New Zealand	2009
*Hanseniaspora*	*uvarum*	NZ234		Grape must	New Zealand	2009
*Hanseniaspora*	*uvarum*	PMH-15-56		Unknown	Lituania	2015
*Hanseniaspora*	*uvarum*	RNH-15-14		Unknown	Lituania	2015
*Hanseniaspora*	*uvarum*	RWH-15-1		Unknown	Lituania	2015
*Hanseniaspora*	*uvarum*	S246-OA		Cherry	Germany (Württemberg)	2014
*Hanseniaspora*	*uvarum*	S382-CB		Grapes	Germany (Württemberg)	2014
*Hanseniaspora*	*clermontiae*	Y-27515^T^	CBS 8821^T^	Rotten stem of a lobelioid plant	Hawaï, United States	2003
*Hanseniaspora*	*jakobsenii*	CBS 12942^T^		Bandji, a traditional palm wine of the palm tree *Borassus akeassii*	Burkina Faso	<2012^∗^
*Hanseniaspora*	*lachancei*	Y-27514^T^	CBS 8818^T^	Fermenting agave juice	Mexico	<2000^∗^
*Hanseniaspora*	*meyeri*	Y-27513^T^	CBS 8734^T^	Fruit of Sapindus sp.	Hawaii Island	<2003^∗^
*Hanseniaspora*	*nectarophila*	CBS 13383^T^		Flower of Siphocampylus corymbiferus	Brazil	2006
*Hanseniaspora*	*singularis*	CBS 10840^T^		Flower	Thailand	<2009^∗^
*Hanseniaspora*	*thailandica*	CBS 10841^T^		Lichen	Thailand	<2009^∗^
*Hanseniaspora*	*valbyensis*	Y-1626^T^	CBS 479^T^	Soil	Danemark	1912
*Kloeckera*	*hatyaiensis*	CBS 10842^T^		Roots of Chamaedaphne calyculata, from rotted wood	Thailand	<2009^∗^

*^∗^Isolation date is anterior to publication or sequence deposition related to this strain.*

### DNA Extraction

DNA extractions were carried out on cells grown in complete medium to stationary phase with two different methods: extraction with the Masterpure yeast DNA purification kit (Epicentre, France), and with an in-house protocol involving a mechanical and chemical lysis. Briefly, cells were resuspended in 200 μL of lysis buffer (Tris 10 mM pH8, EDTA 1 mM, NaCl 100 mM, Triton 2% and SDS 1%), 200 μL of phenol chloroform isoamyl alcohol 25:24:1, and 0.3 g of glass beads. This step was followed by 4 min of vortexing and 5 min of centrifugation at 13,000 rpm. Then, the aqueous phase was mixed with ethanol and the precipitated DNA pellet washed twice with ethanol 70%, dried and resuspended in 100 μL of TE with 1 μL of RNase A at 10 mg/mL.

### Amplification and Sequencing of D1D2 and ITS Regions

All strains, which did not originate from international collections, were identified by amplification and sequencing of the D1D2 and ITSs regions of the ribosomal subunit. The identification of strains from international collections was verified likewise only when there was a doubt about the initial identification after MLST results. Sequences obtained were compared to reference strains by basic local alignment search tool (BLAST) on the YeastIP server^1^. The primers used for these amplifications were ITS1 (TCCGTAGGTGAACCTGCGG) and NL4 (GGTCCGTGTTTCAAGACGG). Amplifications were carried out by polymerase chain reaction (PCR) with the kit Taq Mix (Dongsheng Biotech II) in a 40 μL reaction volume with a first step of DNA denaturation at 94°C for 3 min, followed by 30 cycles of DNA denaturation at 94°C for 30 s, primer hybridization at 55°C for 30 s, and elongation at 72°C for 1 min. A final elongation was performed at 72°C for 3 min. All PCR reaction were performed in a SimpliAmp^TM^ thermal Cycler (Applied Biosystems^TM^).

### Multilocus Sequence Typing

Five housekeeping genes previously used for MLST studies in other yeast species (Bougnoux et al., 2002; Munoz et al., 2009) were selected: ACC1, ADP1, GLN4, RPN2, and VSP13 (Supplementary Table S1). For each marker, homologous sequences of *H. guilliermondii, H. opuntiae* and *H. uvarum* were retrieved from available genomes (Sternes et al., 2016; Seixas et al., 2019) and aligned with Multalin (Corpet, 1988). A set of 11 primer pairs were designed in regions highly conserved surrounding variable sequences of 200–1100 nucleotides. Three additional markers were chosen in intergenic regions of *H. uvarum* genome using Artemis (Rutherford et al., 2000) as a visualization tool: between the homolog of MET5 and the upstream gene, between homologs of SKI2 and DUS3, and between RNR2 and CRM1. Then, homologous sequences of *H. guilliermondii*, and *H. opuntiae* were extracted from EMBL files with Artemis and aligned with that of *H. uvarum* using Multalin. Five primer pairs were designed in these regions the same way as for housekeeping genes (Supplementary Table S1). Selection of the best primer pairs was performed on an initial set of four strains of *Hanseniaspora*: *H. uvarum* CLIB 303^T^, *H. opuntiae* MUCL 49139^T^, *H. guilliermondii* CLIB 510^T^, and *H. pseudoguilliermondii* CLIB 1441. Markers of these strains were amplified and sequenced with each pair of primers. The PCR conditions in a 40 μL reaction volume were: 3 min at 94°C followed by 30 cycles of 30 s at 94°C, 30 s at 55°C, and 1 min at 72°C, with a final step of 2 min at 72°C. Agarose gel electrophoresis was performed to select the best primer pairs. To this end, two criteria were used: (1) amplicon of good intensity in the four strains, (2) a single band per amplicon. Among the 16 primer pairs tested, five were retained and additional pairs of primers were designed specifically to amplify the selected markers in the divergent strain Y-1626 of *H. valbyensis*, considered as the outgroup for phylogenetic trees. In total, 107 strains of *Hanseniaspora* were amplified and sequenced likewise with each of the five selected primer pairs, in order to detect heterozygous sites, to count polymorphic sites inside each species, and to build phylogenetic trees. Sequences of homologous genes in *H. guilliermondii* UTAD222 and *H. uvarum* AWRI 3580 were added to the alignments. For each marker sequence, heterozygous sites were looked for on Chromas (Technelysium)^2^, and replaced by the corresponding degenerated base. Resulting sequences were aligned with clustal, manually cleaned for complex regions with gaps and highly variable positions, and then concatenated using Seaview (Gouy et al., 2010).

For phylogenetic analysis at the species level, concatenated sequences were converted into bi-allelic sequences using an in-house python script. Phylogenetic trees were constructed by maximum likelihood using phyML (Guindon et al., 2009) with a GTR substitution model. Robustness of the tree was assessed by the approximate likelihood ratio test approach (aLRT) and bootstrap of 100 replicates.

### Population Structure Analysis

The concatenated sequence file for the five MLST markers was converted into a STRUCTURE (Pritchard et al., 2000) compatible format with an in-house Perl script where each base is encoded by two digits with values between 0 and 3, allowing consideration of bi-allelic positions. Then, population structure was analyzed with STRUCTURE and InStruct (Gao et al., 2007). Ten runs (STRUCTURE) and 15 runs (InStruct) were performed and the best partitioning was determined for each software output from different criteria: the best likelihood, the increase of likelihood, and the variation between the runs as proposed by CLUMPAK (Kopelman et al., 2015), and using the best DIC (Deviance information criterion) for Instruct. The most frequent consensus partition was then inferred at the optimal number of groups K with CLUMPAK.

### Analysis of Genetic Diversity

Nucleotide diversity was compared between each marker at the species level using the statistics Π and π (Nei and Li, 1979). For that purpose, non-concatenated marker sequences were turned into bi-allelic sequences and analyzed with the R package PopGenome (Pfeifer et al., 2014). In order to infer the mating system, selfing rates were estimated for each species from the Fis obtained with the R package Genepop v1.13 after conversion of the structure data file to the genepop format using PGDspider (Lischer and Excoffier, 2012), and using the RMES software (David et al., 2007) from the heterozygosity profile obtained with a custom script.

### Flow Cytometry

For flow cytometry analysis, cells were first grown overnight in YPD medium at 28°C under 180 rpm agitation, and then diluted to OD_600_ 0.1 in 10 mL YPD and placed in the same conditions during 5 h. After OD measuring, about 10^7^ cells were centrifuged 1 min at 10,000 rpm with 1 mL water. The cell pellets were then suspended in 1 mL water, added drop by drop in 8 mL of ethanol 75% with permanent vortexing and finally stored at 5°C. After one night at 5°C, the cells were centrifuged 5 min at 3,000 rpm, suspended in 1 mL of PBS buffer and centrifuged once again 1 min at 13,000 rpm. Cell pellets were then suspended in 500 μL of RNase A (2 mg/mL in 10 mM Tris-Cl and 15 mM NaCl) and incubated 1 h at 37°C. Finally, cells were treated 1 h at 50°C with 200 μL of 1 mg/mL of proteinase K diluted in PBS buffer, centrifuged and suspended in 500 μL of PBS buffer before being sonicated 15 s in a Branson Sonifier 250 sonicator at 10% of the maximum power. About 10^6^ cells were labeled with SYTOX^®^ green (Invitrogen) in 250 μL at a final concentration of 1 μM. The DNA content was determined on a C6 Accuri (Ann Arbor, MI, United States) spectrophotometer with an excitation wavelength of 488 nm and an emission wavelength of 530 ± 15 nm. Acquisition was performed on 30,000 events observed with a gating on forward scatter/side scatter signal. The flow rate was set to approximately 2,000 events per second (medium flow, 35 μL/min; core, 16 μm). The Python (v. 2.7.13) module FlowCytometryTools (v. 0.5.0) was used for data extraction and manipulation and an “in house” R (v. 3.3.3) script was developed for graphical representations.

### Karyotyping

Yeast karyotyping was achieved by contour-clamped homogeneous electric field (CHEF) gel electrophoresis. Plugs of yeast chromosomes were prepared according to (Vezinhet et al., 1990). The CHEF-DR III pulsed-field gel electrophoresis system (Bio-Rad, Hercules, CA, United States) was set, for a first karyotype, to 3 V/cm with a switching time of 360 s for 23 h and then to 6 V/cm with pulses of 70–90 s for 20 h in 0.5X TBE buffer at 13.5°C in 1% Seakem GTG agarose (FMC) gel. A second karyotype was made to better separate the high molecular weight chromosomes by setting the apparatus to 3 V/cm with pulses of 480 s for 18.1 h, to 4 V/cm with pulses of 300 s for 24 h and to 6 V/cm with pulses 90 s for 12 h in 0.5X TBE buffer at 13°C. We used *S. cerevisiae* CLIB 112 (=YNN295) chromosomal DNA as a marker. The agarose gels were stained with ethidium bromide (0.5 μg/mL) and washed with water before being visualized under UV.

### Genome Sequencing and Analysis

The DNAs of strains CCY46-1-3, CLIB 3101, DBVPG 5828, CLIB 3263, CLIB 3313, CCY-46-1-3a, and S382-CB were used to construct shotgun 400-bp insert libraries, which were sequenced in paired-end (2 × 150 bp) using the Illumina HiSeq2000 platform (Supplementary Table S2). Sequencing reads were cleaned using Fastp v. 0.19.4 (Chen et al., 2018) and used for retrieving MLST marker sequences and whole genome analysis.

First, the cleaned reads of the six interspecific hybrids were used for mapping against the five amplified markers of strains CLIB 1441 (*H. pseudoguilliermondii*) and MUCL 49139^T^ (Type strain of *H. opuntiae*) using HiSat2 v. 2.0.4 (Kim et al., 2015) and the samtools package v. 1.9 (Li et al., 2009). SNPs and Indels were visualized using Artemis to determine manually the sequence of each allele. The different haplotypes obtained in this way were finally considered for the MLST analyses. Second, the cleaned reads were used to estimate the proportion of each parental species using SppIDer (Langdon et al., 2018). This tool only requires the genome sequences of each parental species, in FASTA format, and the sequencing reads of hybrid strains. It is rooted on read mapping and provides various statistics and graphical representations of read coverage against the considered parental genomes. The genome assembly of *H. pseudoguilliermondii* ZIM213 (Shen et al., 2018) and *H. opuntiae* AWRI 3578 (Sternes et al., 2016) were used as references. In order to ease the reading and the interpretation of SppIDer analyses, the scaffolds of these two genomes were preliminary re-ordered to make their sequences collinear as much as possible. To do so, we used MUMmer tool suite v4,0,0beta (Marcais et al., 2018). Maximal unique matches were retrieved with nucmer and the optimal scaffold order was obtained with the -layout option of mummerplot. The dotplot illustrating the re-ordered genome sequences is available in Supplementary Figure S1 on which we also reported the genomic position of the five MLST markers used in this study.

Genome-wide genotyping of hybrid strains was performed as follows. First, cleaned reads were separately aligned on both aforementioned reference genomes using HiSat2 under no-mixed and no-spliced-alignment options, leading to two SAM/BAM files per strain. Mapped read pairs were extracted from these files with samtools, and ambiguous reads that aligned on both reference genomes were removed using BBMap from the BBTools suite^3^ (Supplementary Table S2). Optical and PCR duplicates were then removed with the MarkDuplicates command from Picard v. 2.9.0^4^. Variant calling at the strain level was finally performed using the HaplotypeCaller from GATK v. 3.7, the Genome Analysis Toolkit (McKenna et al., 2010). At this step, raw SNPs and Indels identified were outputted into genomic VCF files (gVCF).

In order to visualize the level of heterozygosity along both parental genomes, each individual gVCF file (two per strain) was first converted into a VCF file using GATK GenotypeGVCFs tool. Raw SNPs were extracted and submitted to a hard-filtering procedure. SNPs that matched the following criteria were filtered out: QualByDepth (QD) < 5.0, FisherStrand (FS) > 55, StrandOddsRation (SOR) > 2.0, RMSMappingQuality (MQ) < 40, MappingQualityRankSumTest (MQRankSum) <−5.0, and ReadPosRankSumTest (ReadPosRankSum) <−5.0. These cutoff thresholds were chosen after visual inspection of the respective distributions of these annotation fields. The rational here was to find a balance between a global transition/transversion ratio (Ts/Tv) close to 2 – an indicator of an effective false positive cleaning – and minimizing the loss of true biological SNPs. Following the hard-filtering procedure, the final call sets were constituted by retaining only biallelic positions. Heterozygous SNPs were then extracted using SnpSift v. 4.3 (Cingolani et al., 2012) and were quantified within 10-kb sliding windows along reference genomes. For that purpose, bedtools v. 2.27.1 (Quinlan and Hall, 2010) was used for both genome splitting into consecutive windows and SNP counting. The minor allele coverage (in percentage of the total read coverage) at biallelic positions was computed for each hybrid genome on 2-kb non-overlapping sliding windows across *H. opuntiae* reference genome.

Assessment of the genetic distance between hybrid strains relied on a similar genotyping approach as that described above, the only difference being that it was based on a joint variant analysis. Briefly, individual gVCF files obtained from one of the parental genome were first pooled together into a cohort gVCF file using the CombineGVCFs tool from GATK. This cohort file was then converted into a VCF file, and the final SNP call set was constituted as previously described. Only an extra filtering step was added in order to remove all positions showing missing data. This cohort call set was first used to estimate nucleotide divergence between strains with the help of Plink v. 1.9 (Purcell et al., 2007) in order to quantify the number of SNPs sharing 0 and 1 Identity By State (IBS0 and IBS1, respectively) in pairwise comparisons for all strains. Principal component analyses were performed on cohort SNP data of each sub-genome using the R package adegenet v. 2.1.1 (Jombart, 2008; Jombart and Ahmed, 2011).

In order to estimate the level of heterozygosity in *H. uvarum* triploid strain S382-CB, we measured the read coverage at each heterozygous position. For that purpose, we genotyped the strain as described above, using *H. uvarum* strain AWRI 3580 as reference genome (Sternes et al., 2016). Only the hard-filtering procedure was applied to the individual VCF file so that all levels of ploidy were retained in the final individual-level call set. Bi- and tri-allelic positions were manually extracted according to the content of the GT and AD fields. Briefly, all positions enclosing either a 0/1- or a 1/2-GT field with less than 5% of total reads supporting the reference allele (AD field) were considered bi-allelic. Conversely, all positions containing a 1/2-GT field with reads supporting both reference and two alternative alleles were considered tri-allelic. Read coverage values were normalized in order to take into account variations of sequencing depth between positions.

### Gene Annotation

As the genome annotation of *H. pseudoguilliermondii* ZIM213 (Shen et al., 2018) was not available, we proceeded to the annotation of protein-coding genes of 576-kb corresponding to the region of CLIB 3263, which showed a 1.4 X coverage compared to the rest of the genome. Non-overlapping open reading frames larger than 180 nt, i.e., with a translated sequence larger than 60 amino acids were compared to *S. cerevisiae* proteome using blastp with an *E*-value threshold of 1.e-10. Sequences without any hit were compared to nr database limited to fungi at the NCBI using blastp. Sequences with positive matches were manually curated for initiator methionine (iMet) and introns. Amino acid sequences with an iMET and smaller than 100 aa without any homologs were discarded. Functional annotations, if any, were transferred from their putative homologs. Similarly, the *H. pseudoguilliermondii* homologous sequence of 126-kb corresponding to the region of *H. opuntiae* lost in CCY 46-1-3a was annotated the same way and manually compared to *H. opuntiae* for synteny and gene model prediction using artemis.

### MAT Locus Analysis

The MAT loci of the reference genomes ZIM213 and AWRI 3578 were annotated (Supplementary Figure S2). Sequencing reads of the hybrid genomes were aligned on reference MAT loci using HiSat2 under no-mixed, no-discordant and no-spliced-alignment options and *k* = 1 parameter. Mapped read pairs were extracted with samtools view and bam2fastq. The recovered reads were then assembled with SPAdes with default parameters (Bankevich et al., 2012).

## Results

### Species Delineation in *Hanseniaspora*

Among 16 primer pairs designed in eight markers, five were selected because they amplified and gave an amplicon of the same size for *H. uvarum*, *H. opuntiae*, *H. guilliermondii*, and *H. pseudoguilliermondii*, as estimated on agarose gel. The selected loci and primer pairs were GLN4 Glutamine tRNA synthetase (GLN4F2/GLN4R2), ADP1 ATP-dependent permease (ADP1F2/ADP1R2), intergenic region upstream of MET5 sulfite reductase beta subunit (MET5F1/MET5R1), RPN2 subunit of the 26S proteasome (RPN2F2/RPN2R2) and VPS13 vacuolar protein sorting (VPS13F2/VPS13R2). 107 strains were successfully amplified and sequenced for MET5, ADP1, RPN2, VPS13 and GLN4, whereas strains of more divergent *Hanseniaspora* species could not be amplified with some primer pairs (Supplementary Table S3). Among the 107 strains, three showed complex chromatograms, characteristic of hybrid markers. After unsuccessful attempts to clone the different alleles due to sequence recombination probably during the PCR step, we finally decided to sequence the whole genomes of CLIB 3101, CCY46-1-3 and DBVPG 5828. Their respective alleles were manually reconstructed by mapping the reads against the five markers of both CLIB 1441 (*H. pseudoguilliermondii*) and MUCL 49139^T^ (*H. opuntiae*). Two to three divergent alleles per marker were obtained for each of the three strains, suggesting that the strains are *H. pseudoguilliermondii* × *H. opuntiae* hybrids. The only exception was an absence of *H. pseudoguilliermondii* MET5 in DBVPG 5828, a region that may have been lost or rearranged.

**FIGURE 1:**
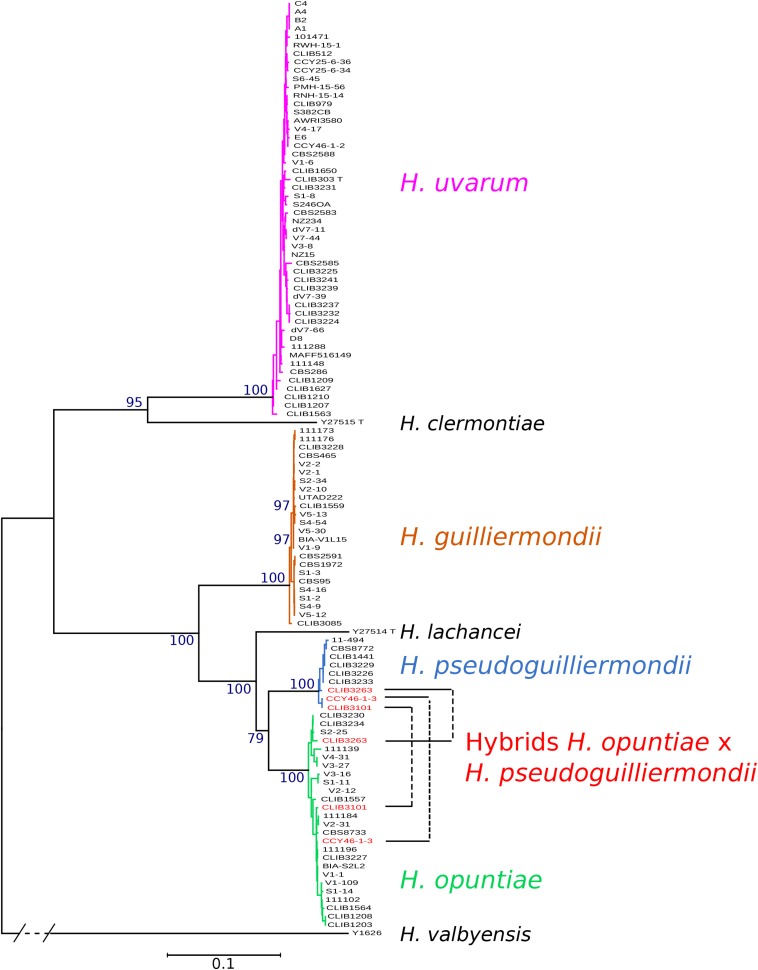
Phylogenetic tree of *Hanseniaspora* strains based on the five marker sequences ADP1, GLN4, RPN2, VPS13, and MET5. The tree was constructed with PhyML based on the concatenated sequence of 3220 sites. DBVPG 5828, which lost *H. pseudoguilliermondii* MET5 allele, was not included in the phylogenetic tree. *H. valbyensis* Y-1626^T^ was used as an outgroup. Branch lengths are proportional to the number of sites that differentiate each pair of strains. Branch support was estimated by the approximate likelihood ratio test approach (aLRT) and bootstrap of 100 replicates.

Phylogenetic trees were constructed from the concatenation of the five markers. Four clearly distinct groups of strains emerged from the tree, corresponding to the four studied species (Figure 1). The topology of the concatenated tree, which included *H. valbyensis* as outgroup and *H. lachancei* and *H. clermontiae* as internal references, was coherent with the topology based on concatenated datasets of actin, D1D2 and ITS gene sequences (Cadez et al., 2006) or on 1,034 orthologous proteins (Steenwyk et al., 2019). As expected, strains of *H. pseudoguilliermondii* appeared as a separate group, very close to the group of *H. opuntiae* strains. Alleles of CLIB 3101, CLIB3263 (its genome was also sequenced, see below) and CCY46-1-3, grouped with both *H. pseudoguilliermondii* and *H. opuntiae* strains, confirming that these strains are hybrids that derive from a cross between the two most closely related species, *H. pseudoguilliermondii* and *H. opuntiae*.

### Population Structure in *H. uvarum*, *H. guilliermondii*, and *H. opuntiae*

In order to explore more thoroughly the phylogenetic relationships between strains of the same species, and to find putative links between strains of the same substrate or geographical origin, we drew trees relying on the concatenated marker sequences, species by species for *H. uvarum, H. guillermondii* and *H. opuntiae*. *H. pseudoguilliermondii* was excluded from this analysis due to the insufficient number of strains. For *H. uvarum* (Supplementary Figure S3) and *H. opuntiae* (Supplementary Figure S4), the tree topology was generally supported by low bootstrap values and no clear correlation to substrate origin emerged. Nevertheless, for *H. uvarum*, a somehow clear phylogenetic signal was obtained for a few strains isolated from the French Guyana and for a group enriched in strains from La Réunion Island, which separated from the other isolates. This signal was echoed by the population structure analysis performed with STRUCTURE and InStruct that provided the same clustering for K equal to three. In contrast, the phylogeny of *H. guillermondii* presented two distinct populations and a single separate strain, CLIB 3085 from Ivory Coast, which attests of the presence of a probable third population (Supplementary Figure S5A). STRUCTURE and InStruct analysis confirmed the partitioning of the strains into three distinct populations (Supplementary Figure S5B). Interestingly, three strains, which were isolated in the same grape must in Ouveillan (Occitanie, France), belong to population 1 (S4–54) or population 2 (S4–16 and S4–9). Similarly, V5–13 and V5–30 belong to population 1 and V5–12 to population 2; all of them were isolated in Pech Rouge (Occitanie, France). Last, for *H. opuntiae*, STRUCTURE and Instruct analyses did not provide any convergent population structure in agreement with the MLST data (Supplementary Figure S4).

### *Hanseniaspora* Species Exhibit Differences in Nucleotide Variability

Analysis of heterozygous and polymorphic sites permitted us to estimate the relative divergence of markers within each species (Table 2). Whereas the most conserved marker was GLN4 in all species, the intergenic region MET5 displayed the highest diversity with up to 10.24% of polymorphic sites and a π value up to 40.55 nucleotides per site in *H. opuntiae*. In this species, MET5 alleles showed two different sizes differing by an insertion of five nucleotides in the intergenic region; some strains showed the presence of both types. Insertions of 3–4 nt were also found in MET5 from *H. guilliermondii* CLIB 3085 and some strains of *H. pseudoguilliermondii*. We also found an insertion of 3 nt in the coding sequence of RPN2 in *H. uvarum*, which corresponds to an additional amino acid. From these data, we could also observe differences between species. *H. opuntiae* showed the highest percentages of polymorphic sites and π values in all markers but GLN4, even if the number of strains studied was two times less than in *H. uvarum.* In contrast, *H. guilliermondii* appeared the least variable species, with values quite similar to that of *H. pseudoguilliermondii* for which we studied three times fewer strains.

**TABLE 2:** Divergence of markers in the four species of *Hanseniaspora.*

	***H. uvarum***					***H. guilliermondii***					***H. opuntiae***					***H. pseudoguilliermondii***				
**MLST locus**	**ADP1**	**GLN4**	**MET5**	**RPN2**	**VPS13**	**ADP1**	**GLN4**	**MET5**	**RPN2**	**VPS13**	**ADP1**	**GLN4**	**MET5**	**RPN2**	**VPS13**	**ADP1**	**GLN4**	**MET5**	**RPN2**	**VPS13**
# Sequences	50	50	50	50	50	24	24	24	24	24	26	26	26	26	26	9	9	9	9	9
# total sites	445	559	909	951	566	440	556	917	973	563	419	564	859	961	551	432	556	918	953	561
# heterozygous sites^1^	10	16	54	50	21	0	2	0	5	0	21	8	80	59	33	4	5	0	1	2
# polymorphic sites^2^	17	21	59	60	24	7	4	21	11	5	22	8	88	60	35	5	5	16	14	5
% polymorphic sites	3.82	3.76	6.49	6.31	4.24	1.59	0.72	2.29	1.13	0.89	5.25	1.42	10.24	6.24	6.35	1.15	0.90	1.74	1.47	0.89
Nucleotide diversity (Π)^3^	4.35	2.87	14.52	17.33	10.00	2.52	1.79	11.12	6.53	3.37	4.62	2.21	40.55	16.9	16.76	3.39	4.11	9.89	10.89	3.33

*^1^The number of heterozygous sites refers to the number of positions where at least one strain has a heterozygous position. ^2^The number of polymorphic sites refers to the number of positions where at least two alleles are present in the strain population, including heterozygous positions. ^3^Nucleotide diversity is measured by the average number of nucleotide differences between all pairs of strains in each alignment.*

As the analysis of the genetic variation in each gene revealed high proportions of heterozygous loci variable across species, we wondered if these differences might originate from different life styles. The estimation of the different allelic frequencies revealed a highly significant deficit of heterozygosity for the four species. However, in relation with the Fis value of each population that varied from 0.1 to 1, the estimate of the selfing rates s varied from 0.21 for *H. opuntiae*, 0.61 for *H. uvarum*, 0.74 for *H. pseudoguilliermondii* and 0.99 for *H guillermondii* (Table 3). In order to avoid the impact of hidden population structure, we estimated Fis and s from a subset of *H. uvarum* devoid of strains from Guyane or la Reunion and we obtained similar results indicating that population structure does not explain Fis and s value for *H uvarum*. Last, selfing rates inferred from the heterozygosity profile with RMES varied also in similar proportions with those obtained from Fis.

**TABLE 3:** Statistics of genetic diversity applied to *Hanseniaspora* sequences.

	***H. uvarum***	***H. guilliermondii***	***H. opuntiae^∗^***	***H. pseudoguilliermondii^∗^***
# Individuals	50	24	23	6
# Polymorphic sites	181	48	213	29
Nucleotide diversity (π) per kb	14.31	7.34	24.16	9.24
Fis	0.4372	0.974	0.116	0.5825
Deficit of heterozygosity *P-*value reject null hypothesis	0.000	0.000	0.000	0.000
Selfing estimated from Fis	0.61	0.99	0.21	0.74
Selfing estimated from heterozygosity profile	0.63 ± 0.08	1.00	0.45 ± 0.08	0.80 ± 0.14

*^∗^The three interspecific hybrids were removed from this analysis.*

One major feature is the complete absence of heterozygous sites in all strains of *H. guilliermondii* but one, CLIB 3085, which contrasts with the substantial heterozygosity of the other species. In order to check if these differences in heterozygosity might be due to differences in ploidy, we analyzed the ploidy of the strains of our strain set.

### Variable Strain Ploidy in *Hanseniaspora* Species

**FIGURE 2:**
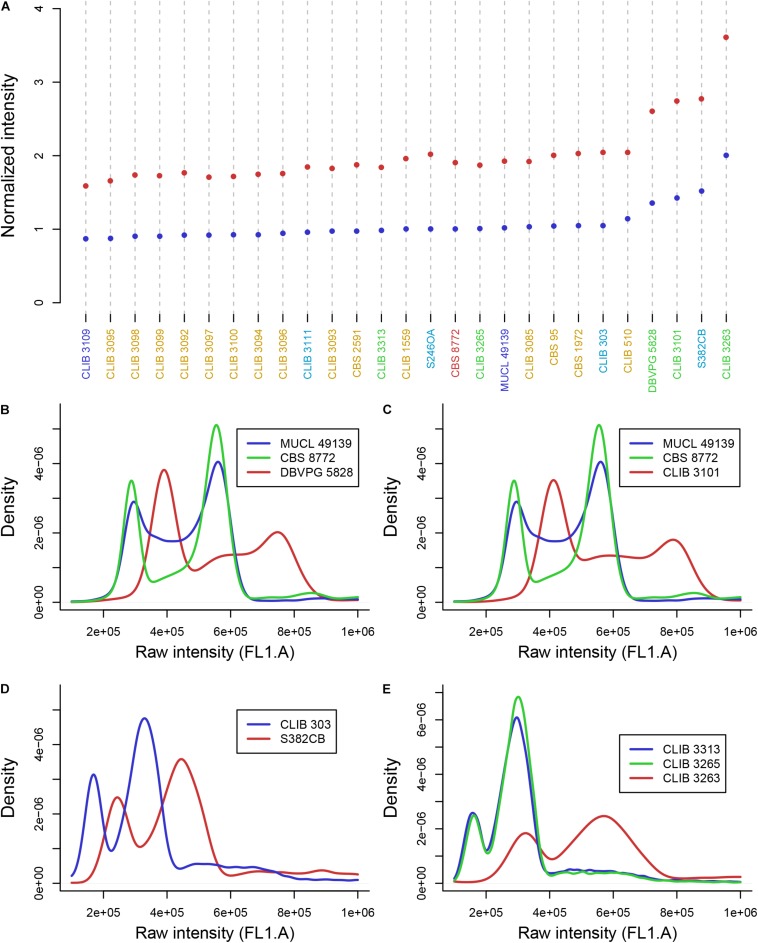
DNA content of *Hanseniaspora* strains as measured by flow cytometry. **(A)** Mean intensity of cell DNA at G1 (blue dots) and G2 (red dots) phases, normalized by the intensity of CBS 8772. Species name are colored in blue (*H. opuntiae*), orange (*H. guilliermondii*), turquoise (*H.* uvarum), green (hybrids), dark red (*H. pseudoguilliermondii*). **(B,C,E)** Intensity curve of *H. opuntiae* × *H. pseudoguilliermondii* hybrids (red) compared to the type strain of the parental species *H. opuntiae* in blue and *H. pseudoguilliermondii* in green. **(D)** Intensity curve of two *H. uvarum* strains, the type strain CLIB 303 and the triploid strain S382CB.

We used flow cytometry and compared the level of Sitox green intensity in *H. guilliermondii* cells and in other *Hanseniaspora* strains that were found heterozygous for the MLST markers, and are thus probably diploid. As shown in Figure 2A, the intensity of all *H. guilliermondii* strains is of the same order as diploid strains of other species, suggesting that these strains are also diploid. As for the hybrids, DBVPG 5828 (Figure 2B) and CLIB 3101 (Figure 2C) were clearly found triploid whereas it was more difficult to evaluate the ploidy of CCY46-1-3 (Supplementary Figure S6). We also found another triploid strain, S382-CB, for which only *H. uvarum* alleles were found in MLST. We can suspect that this strain is not an interspecies hybrid but rather a *H. uvarum* triploid (Figure 2D). Considering the quite high number of hybrids that we found randomly, we decided to screen a collection of additional strains with MET5 as a selective marker. We found three candidate strains isolated from the same biological sample, fermented pineapple from Mayotte Island. All of them have *H. opuntiae* and *H. pseudoguilliermondii* MET5 alleles. Two of them are diploid, CLIB 3313 and CLIB 3265, whereas the third one is tetraploid, CLIB 3263 (Figures 2A,E). As for the first hybrids, the genomes of strains CLIB 3313 and CLIB 3263 were sequenced and the deduced MLST marker sequences, identical for both strains, were added to the phylogenetic tree in Figure 1.

### Genomic Structure of Hybrids

**FIGURE 3:**
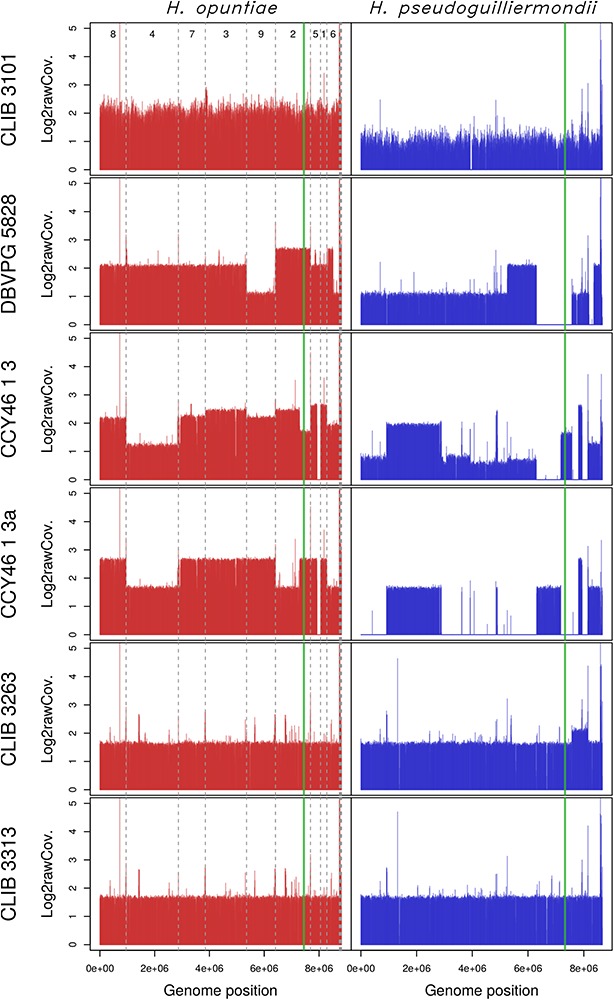
Read coverage from the six hybrid strains along the genomes of the two parental species *H. opuntiae* (in red) and *H. pseudoguilliermondii* (in blue). Reference scaffolds were reordered beforehand so that the genome relative position (*x*-axis) is directly comparable between both parental species. Mean coverage values were computed with SppIDer tool with a sliding-window of 1700 nucleotides (without overlap) and normalized by the mean coverage of *Hanseniaspora* values present in Supplementary Figure S8. Values are expressed in a log2 scale. Dashed gray lines indicate scaffold boundaries. The vertical green line indicates the position of the MAT locus in each subgenome.

Considering that *H. opuntiae* and *H. pseudoguilliermondii* are closely related but distinct species, we hypothesized that they might have different karyotypes as this is the case for sister species in *Saccharomyces* (Fischer et al., 2000) and thus that this could provide us with further clues about the genomic structure of hybrids. However, karyotypes of the type strains of parental species did not indicate any chromosome length polymorphism, and this was the case for the hybrids too (Supplementary Figure S7). We thus decided to sequence their genome with a shotgun strategy with Illumina sequencing chemistry, and to map the reads to reference genomes with SppIDer. To this end, the genome of *H. pseudoguilliermondii* ZIM213 (Shen et al., 2018) and *H. opuntiae* AWRI 3578 (Sternes et al., 2016) were used as references. A preliminary analysis with *H. guillermondii* UTA222, *H. uvarum* AWRI 3580, *H. osmophila* AWRI 3579 and *H. vineae* T02/19AF suggested that the hybrids did not have a third parent (Supplementary Figure S8). The genomes of CLIB 3101, DBVPG 5828 and CCY46-1-3 showed an overall proportion of 2:1, suggesting that these strains are allotriploid *H. opuntiae* (2n) × *H. pseudoguilliermondii* (1n), with the nomenclature proposed by Nguyen and Boekhout (Nguyen and Boekhout, 2017). In CLIB 3101, the reads were homogeneously distributed along the chromosomes whereas in DBVPG5828, differences in parental genome contribution was observed along the chromosomes, accounting for numerous chromosomal rearrangements leading, in some cases, to losses of *H. pseudoguilliermondii* genomic regions, which were counterbalanced by a triploidization of *H. opuntiae* homologous regions (Figure 3). One of the lost regions contained the MET5 allele of *H. pseudoguilliermondii*, which could not be amplified in the MLST approach. The pattern of CCY46-1-3 was much more difficult to analyze as the read coverage sometimes showed intermediate values to 1n, 2n, or 3n. Together with the flow cytometry pattern, this result suggested that CCY46-1-3 might be a population of cells with different genomic contents. We thus selected four individual colonies from CCY46-1-3. They had the same pattern in flow cytometry, suggesting that they were all tetraploid (Supplementary Figure S5). Sequence analysis of the genome of one of them, CCY46-1-3a, showed large *H. opuntiae* genomic regions of probable ploidy 2n and 4n if we consider CCY46-1-3a is a tetraploid. Homologous regions originated from the *H. pseudoguilliermondii* parent were 2n or lost, respectively. A region of 126.5 kb long was lost in *H. opuntiae* and is 4n in *H. pseudoguilliermondii*. One extremity of this region corresponds to the extremity of the scaffolds in both reference genomes (PPNX01000020 in ZIM213; LPNL01000005 in AWRI 3578), which might be a subtelomeric region. The other extremity is internal to the scaffolds, at the locus of homologs of the tandem genes *CSH2* and *CSH3*. Other changes in read coverage occurred internal to *H. opuntiae* scaffolds (e.g., in LPNL01000002, LPNL01000007, and LPNL01000008), which may suggest chromosomal rearrangements rather than chromosome loss and gain.

Sequencing of strains CLIB 3263 and CLIB 3313 showed the same proportion of sequences from *H. opuntiae* and *H. pseudoguilliermondii* parents, with the exception of a *H. pseudoguilliermondii* region of about 576 kb in CLIB3263, which had a coverage ratio of about 1.4 compared to the rest of the genome. Knowing that CLIB 3263 is tetraploid, this suggests that either a segmental or a chromosome duplication occurred in only one of the two *H. pseudoguilliermondii* homologous chromosomes – but in that case the ratio should be 1.5, not 1.4 – or that CLIB 3263 is a population of heterogeneous cells having undergone or not an event of segmental or chromosome duplication.

### Genetic Diversity of Hybrids

To get clues about the event that led to the hybrid formation, we first investigated their MAT loci by comparison to those of reference genomes. *H. pseudoguilliermondii* ZIM 213 has a single MAT locus with only MATalpha1 gene. To recover the MATa locus we used CBS 8772, which is diploid and possesses both MAT loci. The reference genome of *H. opuntiae* AWRI 3578 contains a MAT locus with both MATalpha1 and MATa2 genes (Supplementary Figure S2A). Mapping and assembly of the mapped reads of the six hybrids revealed that CCY46-1-3, CCY46-1-3a and DBVPG 5828 had both MATa and MATalpha from *H. opuntiae*, suggesting that the parental diploids were MATa/MATalpha, whereas CLIB 3101 had only a MATa locus (Supplementary Figure S2B). CCY46-1-3 and CLIB 3101 had also a *H. pseudoguilliermondii* MATalpha locus whereas CCY46-1-3a and DBVPG 5828 have lost this genomic region, which has been replaced by its *H. opuntiae* counterpart (Figure 3). CLIB 3263 and CLIB 3313 had a single MAT locus per subgenome, MATa from *H. opuntiae* and MATalpha from *H. pseudoguilliermondii*.

**FIGURE 4:**
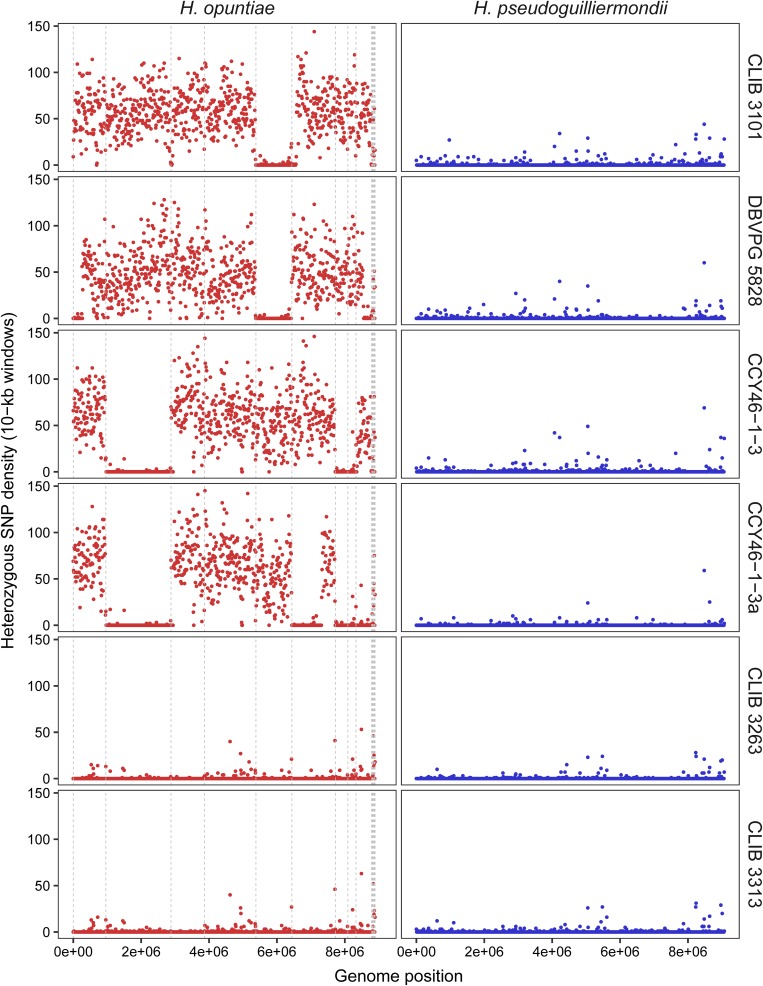
Density of heterozygous SNPs along *H. opuntiae* and *H. pseudoguilliermondii* collinearized parental genomes in hybrid strains. Each dot represents the total number of heterozygous SNPs enclosed within 10-kb sliding windows. Scaffold order of reference genomes is the same as in Figure 3. Dashed gray lines indicate scaffold boundaries.

Then, we investigated the level of heterozygosity in both subgenomes of each hybrid. A high level of heterozygosity, up to 0.503%, was observed for *H. opuntiae* subgenome in strains CLIB 3101, DBVPG 5828, CCY46-1-3 and its derivative CCY46-1-3a, which suggests that *H. opuntiae* parental strains were heterozygous diploids in each case (Table 4). This level is probably under-estimated, as only bi-allelic positions in the population were considered. The distribution of heterozygous positions was not homogeneous along the chromosomes (Figure 4 and Supplementary Figure S9), and showed some regions of loss of heterozygosity (LOH). Interestingly, the largest LOH in CLIB 3101 and DBVPG 5828 covers the same region. In contrast, in these four strains a very low level of heterozygosity was observed for the *H. pseudoguilliermondii* subgenomes, even in the duplicated or triplicated chromosomal regions, which may correspond to false positive, sequencing errors or single nucleotide mutations after duplication. In strains CLIB 3263 and CLIB 3313, very few heterozygous sites were observed, neither for *H. opuntiae*, nor for *H. pseudoguilliermondii* subgenomes. This is coherent with the fact that CLIB 3313 is a diploid with 1n chromosomes from each parent and this suggests that the tetraploid strain CLIB 3263 derives from an auto-diploidization event. This also corroborates the subsequent 576-kb segmental duplication event in CLIB 3263.

**TABLE 4:** Percentage of heterozygosity in *H. opuntiae* and *H. pseudoguilliermondii* subgenomes of hybrids.

**Strain**	***% H. opuntiae***	***% H. pseudoguilliermondii***
DBVPG 5828	0.406	0.007
CCY46-1-3	0.424	0.009
CLIB 3101	0.503	0.009
CCY46-1-3a	0.365	0.003
CLIB 3263	0.008	0.004
CLIB 3313	0.009	0.005

**FIGURE 5:**
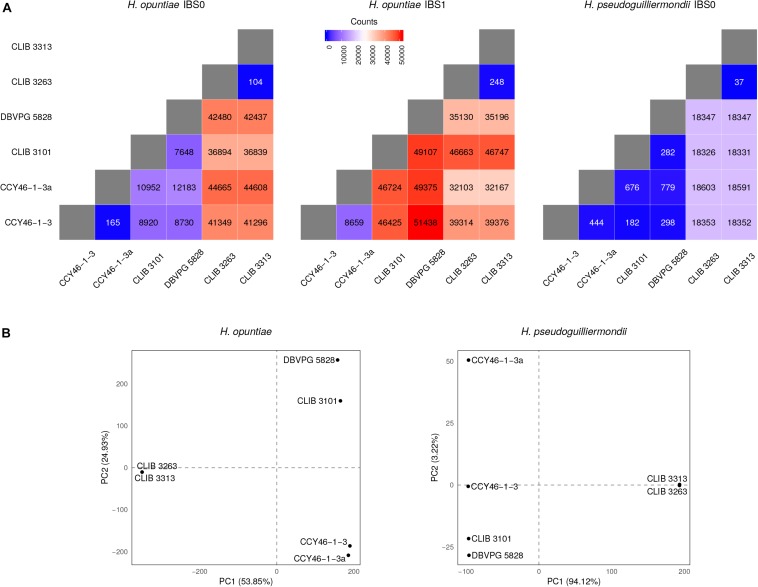
Analysis of individual-based genetic distance between hybrid strains. **(A)** Heatmap representations of the number of differentiating SNPs between strain pairs. The divergence between strains was assessed here using the number of IBS0 and IBS1 (Identity By State) SNPs. A SNP is defined as IBS0 when no allele is shared within the considered pair, and IBS1 when one allele is found in common. Therefore, as hybrids are haploid on their *H. pseudoguilliermondii* subgenome part (or auto-diploid in the case of CLIB 3263), only IBS0 SNPs were analyzed in that context. **(B)** Principal Component Analyses of the hybrid strain total SNP data. The principal components were constructed as linear combinations of 117,191 and 23,189 total SNPs identified following a joint variant analysis of *H. opuntiae* and *H. pseudoguilliermondii* reference genomes, respectively. Only the two first components are displayed, the total variance supported by both axes is indicated within brackets.

As MLST marker sequences were almost identical in CLIB 3101 and DBVPG 5828 and as their regions of LOH were at the same genomic position, we suspected that both strains could derive from the same hybridization event. To address this hypothesis, we examined the pairwise allele sharing between strains across the genomic regions present in all hybrids, which represents more than 95% of *H. opuntiae* and about 30% of *H. pseudoguilliermondii* reference genomes. It appeared that CLIB 3101 and DBVPG 5828 showed a major divergence regarding their respective *H. opuntiae* subgenomes. There are 7648 positions with no nucleotide in common (IBS0), and 49,107 positions (IBS1) where only one allele is in common (Figure 5A). These data suggest that the parental diploids were different and consequently that CLIB 3101 and DBVPG 5828 may derive from two distinct hybridization events. The *H. pseudoguilliermondii* part is much less divergent with only 282 different positions between the two strains, which may be linked to the low level of divergence observed in *H. pseudoguilliermondii* with the MLST analysis. The same analysis performed on the other interspecific hybrid genomes showed that the three European strains are different between them at least for *H. opuntiae* subgenomes and very distant from the two strains from Mayotte Island for both subgenomes (Figure 5B).

Assessment of the heterozygosity in *H. uvarum* triploid strain S382-CB revealed 55,355 bi-allelic positions with one allele in common with the reference strain AWRI 3580, 160 bi-allelic positions with both alleles being alternative to the reference allele, and 20 tri-allelic positions. Bi-allelic positions presented a distribution of read coverage of 1/3-2/3, which confirm the triploid nature of the strain (Supplementary Figure S10).

## Discussion

The aim of this study was to characterize and to clarify the genetic relationships between strains of a species complex with a method that would facilitate further studies of *Hanseniaspora* strains, especially strains isolated from vineyards. The results obtained from the MLST approach showed that even if five loci are not representative of the whole genome, our markers are pertinent to discriminate species and therefore hybrids. Indeed, the topology of our multi-species tree is consistent with that of Steenwyk et al. (2019) based on 1,034 orthologous groups, with strong bootstrap values. In comparison, trees based on classical taxonomic markers harbor variable topologies sometimes poorly supported. For instance, the trees of Cadez et al. (2006, 2019) showed different topologies due to the additional use of EF-1α in the latter tree. It is therefore essential to have relevant markers that allow resolving accurately species delineation. Our combination of markers is also sufficiently divergent to unveil the presence of subpopulations, as this is the case for *H. guilliermondii* populations. However, we failed to establish a clear population structure related to substrate origin or geographical localization except for *H. uvarum* for which a group of strains isolated from Guyana and a group enriched in strains from La Réunion Island separated from the other strains. Using microsatellite analysis, Albertin et al. (2016) reported some population structure for *H. uvarum* oenological strains according to geographic origin, i.e., South Africa versus other origins, primarily from France and New Zealand, and to sampling year. However, this clustering might be a fuzzy rule, as some Bordeaux isolates such as strain CRB1430 were identical to South Africa isolates.

Another aspect of our MLST analysis is the assessment of the level of genetic diversity according to species and markers. As expected, the four markers designed in exons of housekeeping genes (GLN4, ADP1, RPN2 and VPS13) were generally less divergent than MET5, which includes an intergenic region, known to be highly variable. It was, however, surprising to reach almost 10% of polymorphic sites in *H. opuntiae* MET5 with only 26 studied strains, whereas it is only 2.29% in *H. guilliermondii* with nearly the same number of strains. This clearly denotes a species-specific variability, with a significant difference even in the two most closely related species, *H. opuntiae* and *H. pseudoguilliermondii*. Another interesting result emerged from the level of heterozygosity. No heterozygous sites were observed in strains of *H. guilliermondii*, except in CLIB 3085. This finding does not result from a difference of ploidy, as all of the *H. guilliermondii* strains were found diploid by flow cytometry, like most of the strains from the other species. This rather indicates a major difference in life style compared to the other species. While we observed a high genetic diversity, a high heterozigosity for most species and some population structure with admixture for *H. uvarum*, the absence of heterozygosity suggests an absence of random mating in both population 1 and population 2 of *H. guilliermondii*. A whole genome sequencing strategy at the population level may provide clues to address this hypothesis.

The use of MLST markers compatible with multiple species amplification allowed us to detect interspecific hybrids with a surprisingly high frequency, i.e., six strains among 107 studied strains. Yeast hybrids have been extensively studied in *Saccharomyces* sister species. They appeared to occur rarely in nature but much more frequently in anthropogenic environments, where they present a great interest for their biotechnological potentials in winemaking and lager brewing (Sipiczki, 2018). With the development of second and third generation sequencing technologies, interspecies hybrids have been reported for a number of other Saccharomycotina species, such as *Pichia sorbitophila* (Louis et al., 2012), *Zygosaccharomyces bailii* (Mira et al., 2014; Ortiz-Merino et al., 2017), *Zygosaccharomyces parabailii* (Braun-Galleani et al., 2018), *Zygosaccharomyces rouxii* (Gordon and Wolfe, 2008; Solieri et al., 2008; Bizzarri et al., 2019), *Dekkera bruxellensis* (Borneman et al., 2014), *Candida orthopsilosis* (Schroder et al., 2016), or *Saccharomycopsis fibuligera* (Farh et al., 2017).

**FIGURE 6:**
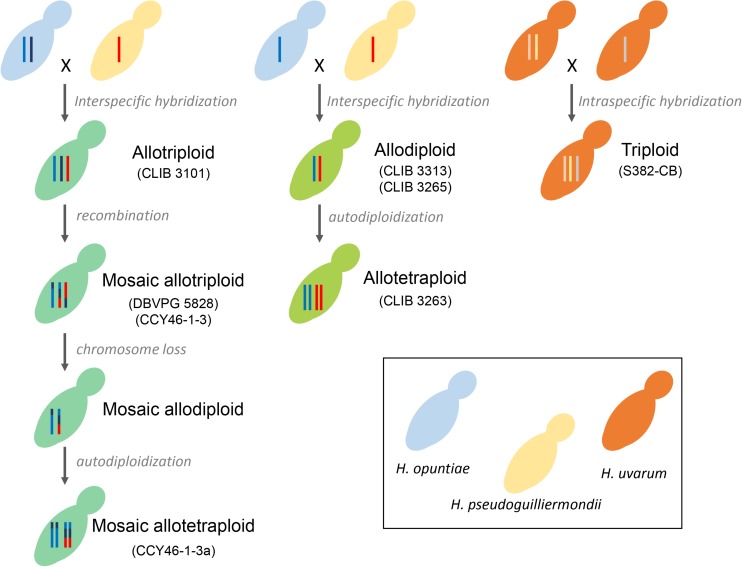
Scenario for hybrid formation. Each *Hanseniaspora* species is depicted by color coded budding cells: blue (*H. opuntiae*), yellow (*H. pseudoguilliermondii*), orange (*H. uvarum*), and different shades of green for the hybrids, depending on the proportion of each parental species. The number of vertical lines inside the mother cells represents ploidy: one line for haploids, two for diploids, three for triploids and four for tetraploids. Lines with multiple colors represent Mosaic chromosomes.

We exclusively found hybrids between the two closest related species *H. opuntiae* and *H. pseudoguilliermondii*. They showed different genomic structures. CLIB 3101, DBVPG 5828 and CCY46-1-3 are allotriploids with different degrees of chimerism. CCY46-1-3a, a single colony of CCY46-1-3, is an allotetraploid. CLIB3313 and CLIB 3263 are allodiploid and allotetraploid, respectively. We propose different scenarios of hybrid creation in Figure 6. Genome sequencing of recently isolated strains CLIB 3101, CLIB 3313 and CLIB 3263 showed an absence of mosaicism between the parental strains. They probably derive from a recent rare mating between a diploid cell of *H. opuntiae* and a haploid cell of *H. pseudoguilliermondii* for CLIB 3101 and from a mating between two haploid cells for CLIB 3313 and CLIB 3263. These scenarios are in agreement with the organization of their MAT loci. Indeed, CLIB 3313 and CLIB 3263 are *H. opuntiae* MATa and *H. pseudoguilliermondii* MATalpha. CLIB 3101 is homozygous MATa/MATa for the mating type of *H. opuntiae* subgenome and MATalpha in *H. pseudoguilliermondii* part, which enables the mating program (Sipiczki, 2018). As there are no silent cassettes in *H. opuntiae*, there are only two ways to become MATa/MATa, i.e., the conversion of MATalpha to MATa in a diploid cell, or auto-diploidization of a haploid MATa strain. As heterozygosity was observed in *H. opuntiae* subgenome, we favored the conversion hypothesis. In contrast, DBVPG 5828 and CCY46-1-3 have complex genomes with multiple chimeric chromosomes and unexpectedly they possess both MATa and MATalpha loci in the *H. opuntiae* subgenome, which suggests more intricate creation and evolution scenarios than in CLIB 3101. Genome sequences and karyotypes of hybrids, which are similar to those of parental species strains, showed that at least some genomic rearrangements occurred by non-reciprocal translocations between chromosomes and replacement of intrachromosomal regions by their homologs from the other subgenomes. Duplication/loss of entire chromosomes may have also occurred but as the reference genomes have not been assembled at the chromosome level (Sternes et al., 2016; Shen et al., 2018), it is therefore difficult to validate it. Both strains have been conserved in collection for 10 years, which addresses the question of the stability and evolution of conserved hybrid strains. Indeed, CCY46-1-3 comprises a population of cells deriving from the initial mating. Isolation of four single colonies showed a unique tetraploid pattern in flow cytometry. Sequencing of CCY46-1-3a showed that, compared to CCY46-1-3, the equivalent of a 1n genome of *H. pseudoguilliermondii* has been lost, leading to a 2n equivalent genome. This implies that the isolate has been further duplicated by auto-diploidization to form the resulting mosaic allotetraploid (Figure 6). Similarly, as the genome of CLIB 3313 and CLIB 3263, which have been isolated in the same pineapple fermentation, were almost identical in sequence, it is likely that CLIB 3263 derives from CLIB 3313 by auto-duplication. Rare mating leading to allotriploid genomes have been widely reported in *Saccharomyces* genus, with for instance *S. cerevisiae* (2n) × *S. kudriavzevii* (1n) hybrid VIN7 (Borneman et al., 2012) or *S. cerevisiae* (1n) x*S. eubayanus* (2n) strains of *S. pastorianus* group I/Saaz-type (Monerawela and Bond, 2017). *D. bruxellensis* is another species example in which AWRI 1499 and AWRI 1608 are *D. bruxellensis* (2n) × *Dekkera sp.x* (1n) and *D. bruxellensis* (2n) × *Dekkera sp.y* (1n), respectively, with *Dekkera sp.x and Dekkera sp.y* two distinct unknown species (Borneman et al., 2014).

Hybridization is known to provide selective advantage in a stressful environment or to cumulate advantageous properties such as the ability to grow at low temperature together with robust fermentation characteristics as reported for the lager yeast *S. pastorianus* (Gibson and Liti, 2015). It is also a mechanism for restoration of fertility (Ortiz-Merino et al., 2017; Watanabe et al., 2017; Braun-Galleani et al., 2018). To get clues about the benefit obtained by *Hanseniaspora* hybrids, we investigated the differences in gene content of particular regions between both subgenomes. Thus, we investigated the *H. pseudoguilliermondii* triplicated 126-kb region in CCY46-1-3a. By comparing reference genomes, we found 68 genes in *H. opuntiae* and 67 homologs in *H. pseudoguilliermondii.* The difference comes from two consecutive genes g2511 and g2512 corresponding to pseudogenes in *H. opuntiae*, which form a single gene in *H. pseudoguilliermondii* encoding the peroxisomal biogenesis factor 6. Whether the replacement of this pseudogene by its functional counterpart was the reason of this triplication will require further experimental data. Similarly, we investigated the 576-kb region that has been duplicated in *H. pseudoguilliermondii* subgenome of CLIB 3263. 312 putative protein-coding genes were annotated, 45 were specific to *Hanseniaspora* genus with unknown function. For the other genes, we considered the function of *S. cerevisiae* homologs, but failed to identify any bias, except a putative overrepresentation of mitochondrial proteins.

## Conclusion

This study provides efficient markers that could be used to identify rapidly species and hybrids from closely related *Hanseniaspora* recovered from grapes and musts. Our approach revealed the presence of some putative population structure in three species, and showed differences in the species lifestyle, which make it an interesting yeast species cluster to explore further, especially in the context of the potential adaptation to the wine environment. As in many other Saccharomycotina yeasts, we have found hybrid genomes in *Hanseniaspora.* The presence of different types and origins of these strains, i.e., allotriploids with different degrees of mosaicism, allodiploids and allotetraploids, is one of the most interesting aspect. Moreover, the genomes seem particularly instable with frequent auto-diploidization. This is therefore a unique model to study the evolution and stability of genomes in a genetic context that do not derives from the whole genome duplication.

## Data Availability Statement

Sequencing reads of *H. opuntiae* × *H. pseudoguilliermondii* hybrids have been deposited at the EMBL-ENA under the accession numbers ERR3456268, ERR3456265, ERR3456264, ERR3456266, ERR3456262, and ERR3456263 (project PRJEB33345).

## Author Contributions

CN conceived the project. MS, CN, and J-LL designed the experiments. MS, CB, CG, MP, and CN performed the experiments. HD, LP, JL-L, MS, and CN performed the bioinformatics analyses. MS and CN wrote the first draft. HD, LP, J-LL, CG, MS, and CN edited the manuscript. All authors read and approved the final version.

## Conflict of Interest

The authors declare that the research was conducted in the absence of any commercial or financial relationships that could be construed as a potential conflict of interest.

## Footnotes

1 http://genome.jouy.inra.fr/yeastip/

2 http://technelysium.com.au/wp/

3 sourceforge.net/projects/bbmap

4 https://broadinstitute.github.io/picard
