# Supplementary material for: Investigation of Genetic Relationships Between Hanseniaspora Species Found in Grape Musts Revealed Interspecific Hybrids With Dynamic Genome Structures
Source: Front Microbiol. 2020 Jan 15;10:2960. doi: 10.3389/fmicb.2019.02960 (PMC6974558; doi:10.3389/fmicb.2019.02960)
Supplement: Supplementary file 1 [file Data_Sheet_1.PDF]

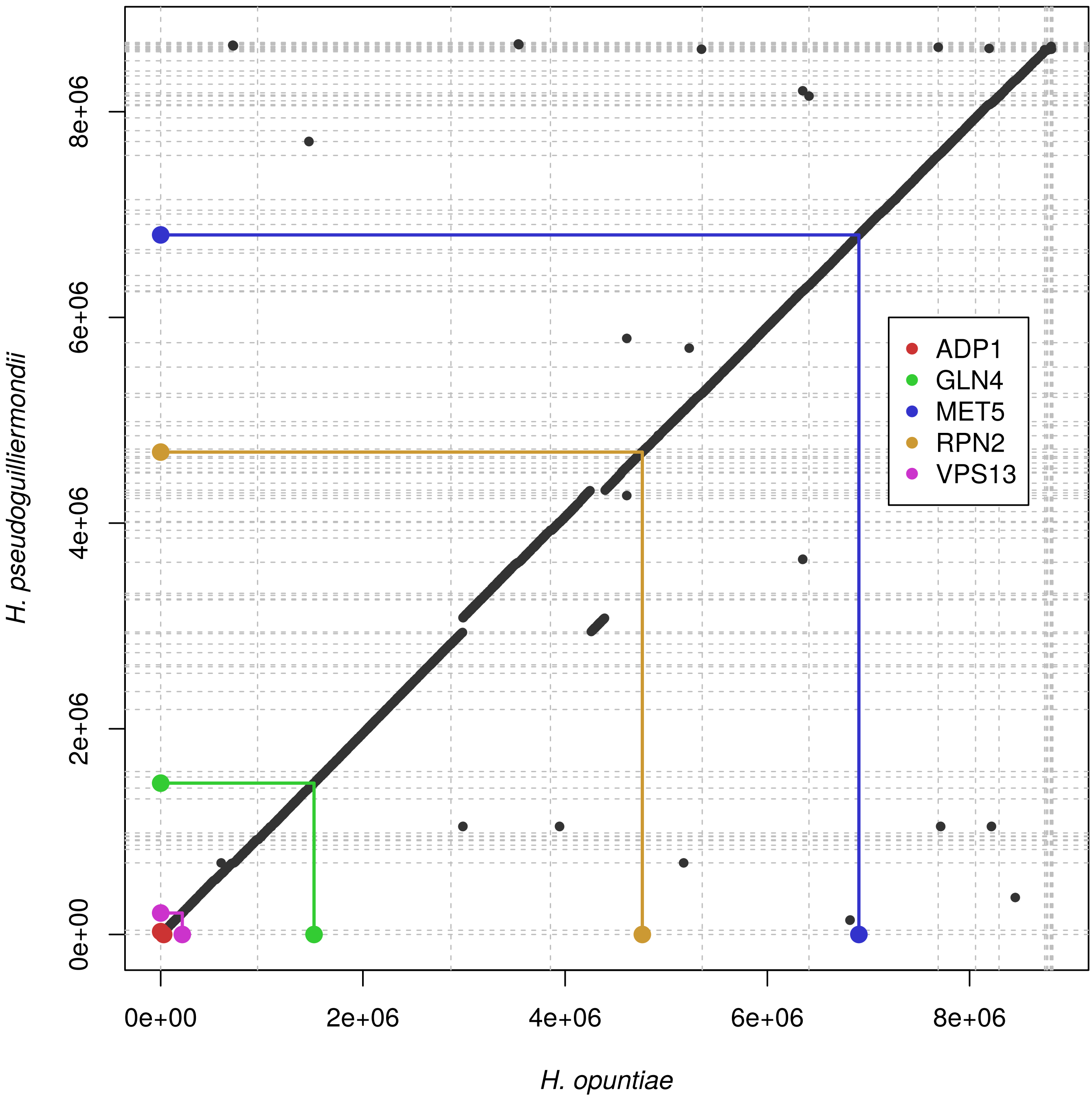

Supplementary Figure S1: Dotplot between the re-ordered genome sequences of *H. opuntiae* and *H. pseudoguilliermondii*. Black dots correspond to maximal unique matches of at least 500 nucleotides between the two genomes. Coloured dots indicate the location of the five MLST markers used in this study.
