# Supplementary material for: Investigation of Genetic Relationships Between Hanseniaspora Species Found in Grape Musts Revealed Interspecific Hybrids With Dynamic Genome Structures
Source: Front Microbiol. 2020 Jan 15;10:2960. doi: 10.3389/fmicb.2019.02960 (PMC6974558; doi:10.3389/fmicb.2019.02960)
Supplement: Supplementary file 2 [file Data_Sheet_2.PDF]

A

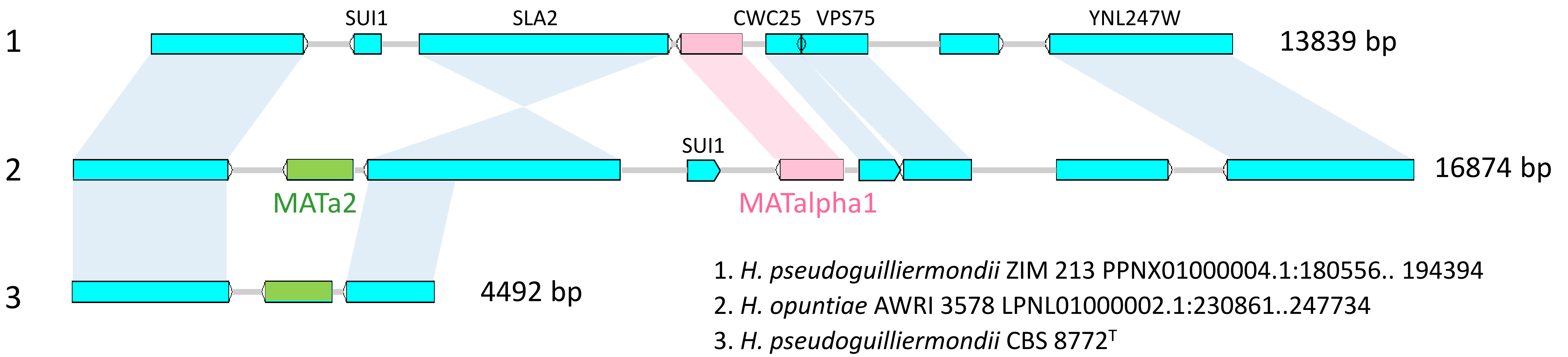

B

	***H. opuntiae* (1)**				***H. pseudoguilliermondii* (2)**			***H. pseudoguilliermondii* (3)**		
	**# Reads**	**# Scaffolds**	**MATa**	**MATalpha**	**# Reads**	**# Scaffolds**	**MATalpha**	**# Reads**	**# Scaffolds**	**MATa2**
CCY46-1-3	5610	7	MATa2	MATalpha1	5836	2	MATalpha1	1315	2*	No
CCY46-1-3a	6939	10	MATa2	MATalpha1	59	5	lost	0	NA	No
DBVPG5828	10272	17	MATa2	MATalpha1	91	8	lost	0	NA	No
CLIB3101	4571	10	MATa2	No	2365	8	MATalpha1	480	2*	No
CLIB3263	4322	1	MATa2	No	4183	1	MATalpha1	861	2*	No
CLIB3313	3849	1	MATa2	No	3975	1	MATalpha1	747	2*	No

*The two scaffolds corresponded to the two genes flanking MATa2, and we did not find any read mapping to MATa2.

Supplementary Figure S2: MAT locus of *H. pseudoguilliermondii* and *H. opuntiae.*
A. Schematic representation of the MAT locus of reference genomes: (1) ZIM 213 MATalpha locus, (2) AWRI3578 MAT*a* and MATalpha loci, and (3) CBS8772 MATa locus. The region carrying SUI1-SLA2-MATalpha1 is inverted compared to its counterpart at the MATa locus. This inversion has been identified in *H. pseudoguilliermondii, H. opuntiae, H. uvarum* and *H. guilliermondii*.
B. Sequencing reads of the 6 hybrids were mapped on the three MAT reference sequences.
#Reads: number of read pairs mapping the reference sequences (locus 1, 2 or 3).
#Scaffolds: number of scaffolds afetr assembly with spades.
