# Supplementary material for: Investigation of Genetic Relationships Between Hanseniaspora Species Found in Grape Musts Revealed Interspecific Hybrids With Dynamic Genome Structures
Source: Front Microbiol. 2020 Jan 15;10:2960. doi: 10.3389/fmicb.2019.02960 (PMC6974558; doi:10.3389/fmicb.2019.02960)
Supplement: Supplementary file 4 [file Data_Sheet_4.PDF]

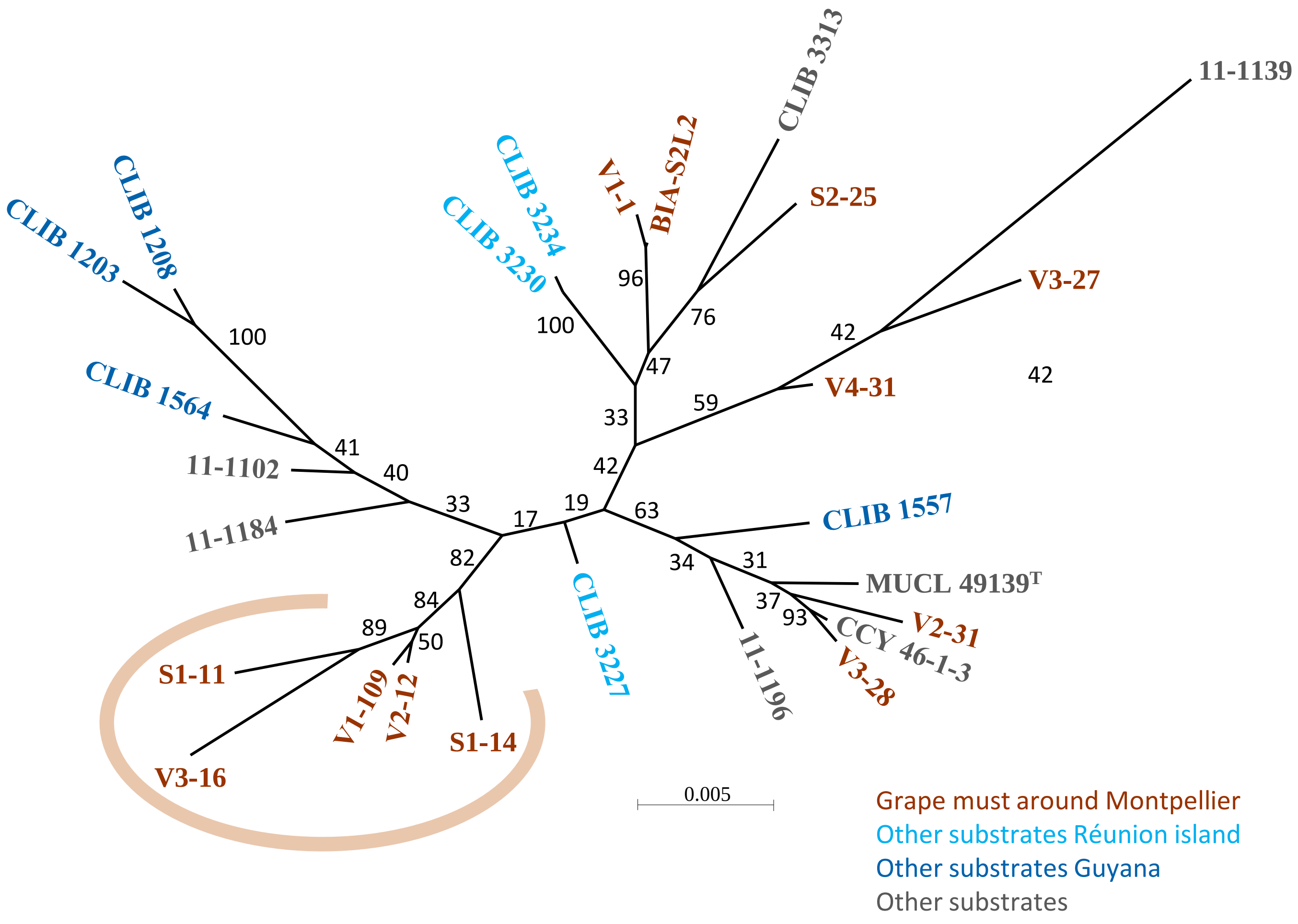

Supplementary Figure S4: Phylogenetic tree of *H. opuntiae* strains. The unrooted tree was reconstructed with PhyML from the alignment of 3352 residues resulting from the concatenation of the five markers. Strain names are coloured according to their geographical and/or substrate origin: Brown for grape musts in Montpellier region (France), turquoise for other substrate in La Réunion Island, blue for other substrates in Guyana, and grey for other substrates in other countries. A well-supported branch of strains isolated from region Occitanie is circled in brown.
