# Supplementary material for: Investigation of Genetic Relationships Between Hanseniaspora Species Found in Grape Musts Revealed Interspecific Hybrids With Dynamic Genome Structures
Source: Front Microbiol. 2020 Jan 15;10:2960. doi: 10.3389/fmicb.2019.02960 (PMC6974558; doi:10.3389/fmicb.2019.02960)
Supplement: Supplementary file 5 [file Data_Sheet_5.PDF]

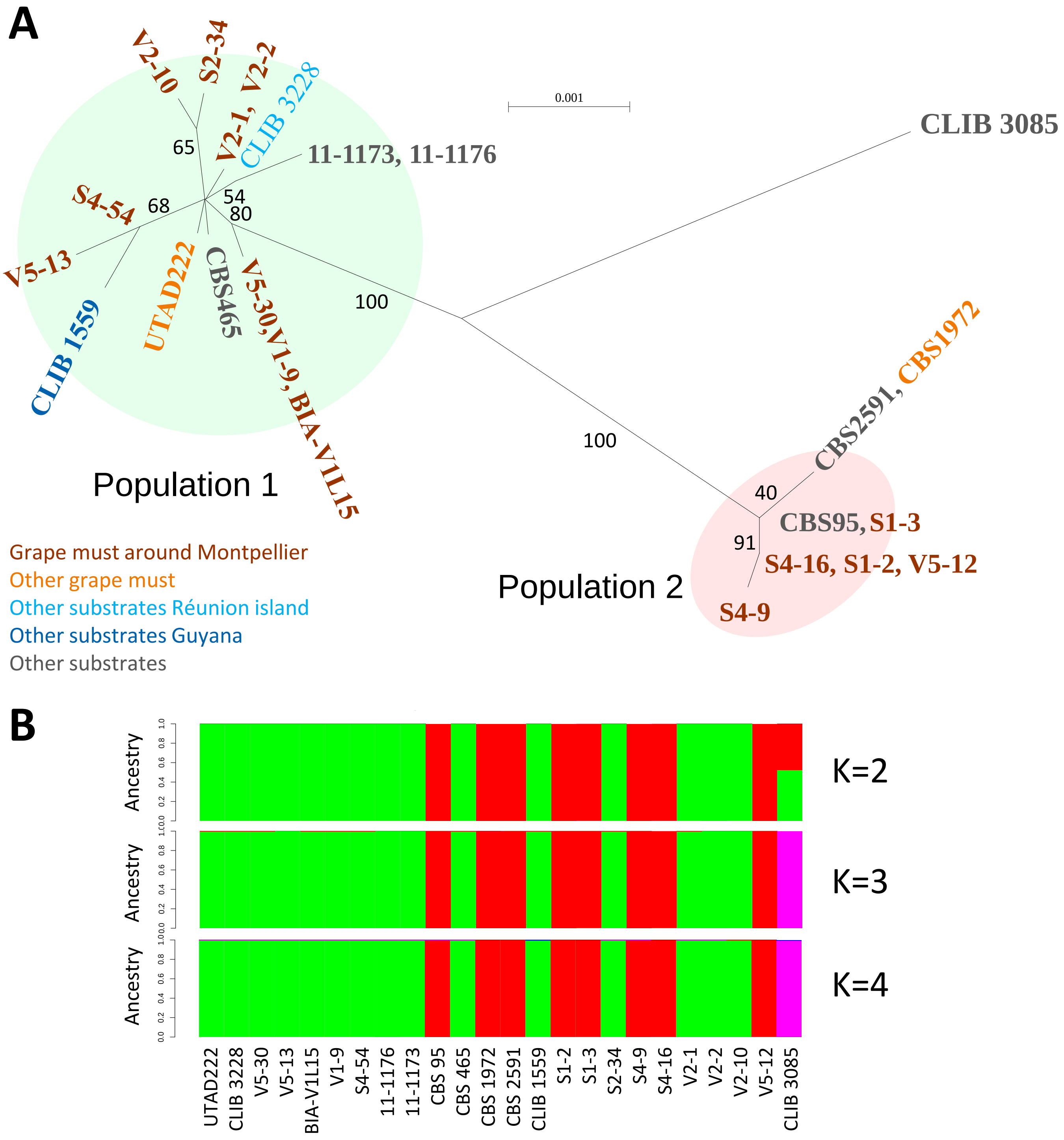

Supplementary Figure S5: Population structure of *H. guilliermondii.* **A**. The phylogenetic tree is based on the concatenation of the five MLST markers. The unrooted tree was reconstructed with PhyML from the alignment of 3449 residues. Strain names are coloured according to their geographical and/or substrate origin: Brown for grape musts in Montpellier region (France), orange for grape musts from other countries, turquoise for other substrate in La Réunion Island, blue for other substrates in Guyana, and grey for other substrates from other countries. The strain group corresponding to population 1 is coloured in light green and in light pink for population 2. **B**. Population structure inference with Structure with K=2 to K=4. Strains with green colour correspond to population 1, and with red colour to population 2.
