# Supplementary material for: Investigation of Genetic Relationships Between Hanseniaspora Species Found in Grape Musts Revealed Interspecific Hybrids With Dynamic Genome Structures
Source: Front Microbiol. 2020 Jan 15;10:2960. doi: 10.3389/fmicb.2019.02960 (PMC6974558; doi:10.3389/fmicb.2019.02960)
Supplement: Supplementary file 6 [file Data_Sheet_6.PDF]

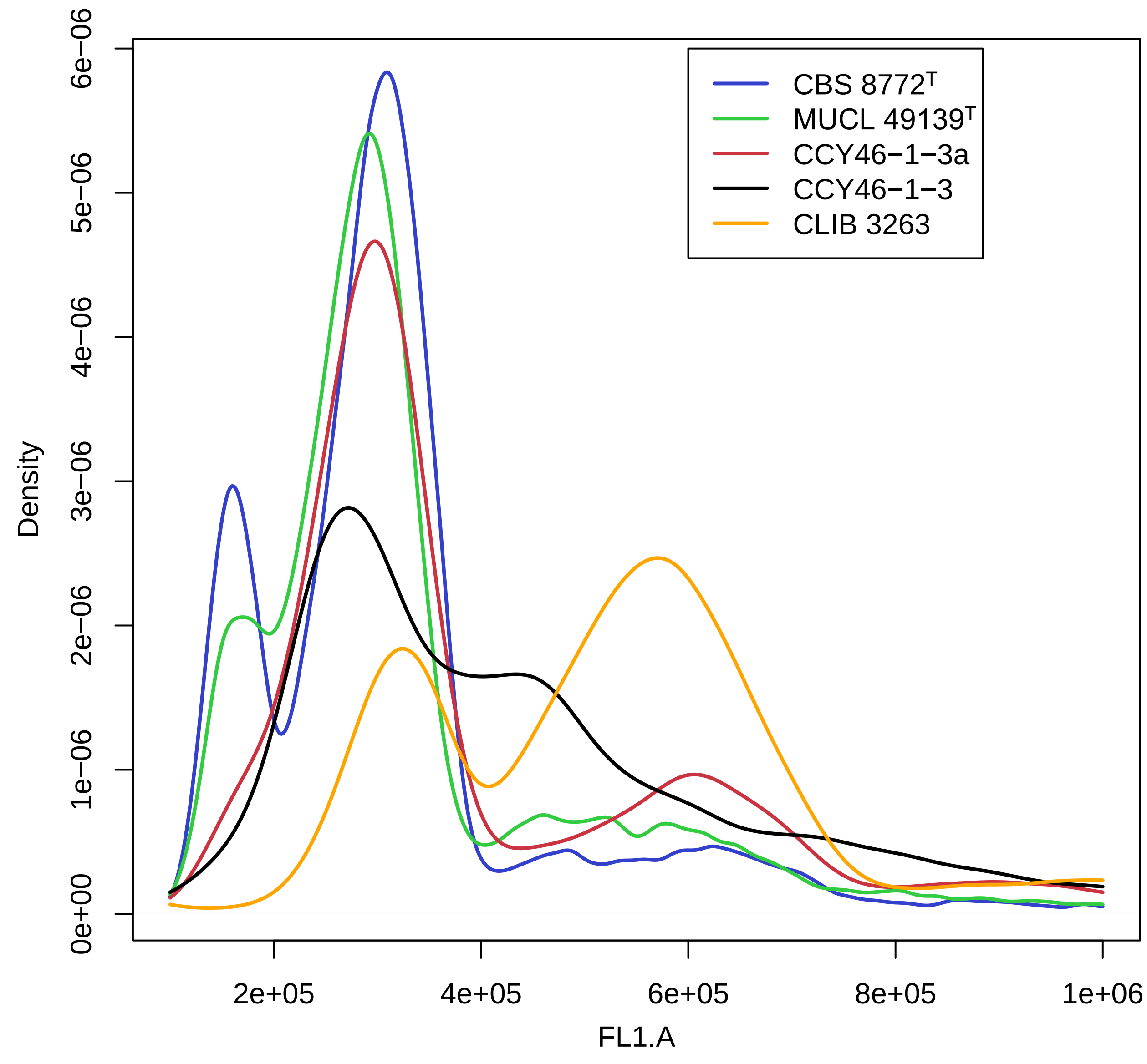

Supplementary Figure S6: Flow cytometry of strain CCY46-1-3 and its derivative CCY46-1-3a compared to diploid strains of *H. opuntiae* MUCL 49139^{T} and *H. pseudoguilliermondii* CBS 8772^{T} and the tetraploid strain CLIB 3263.
