# Supplementary material for: Investigation of Genetic Relationships Between Hanseniaspora Species Found in Grape Musts Revealed Interspecific Hybrids With Dynamic Genome Structures
Source: Front Microbiol. 2020 Jan 15;10:2960. doi: 10.3389/fmicb.2019.02960 (PMC6974558; doi:10.3389/fmicb.2019.02960)
Supplement: Supplementary file 7 [file Data_Sheet_7.PDF]

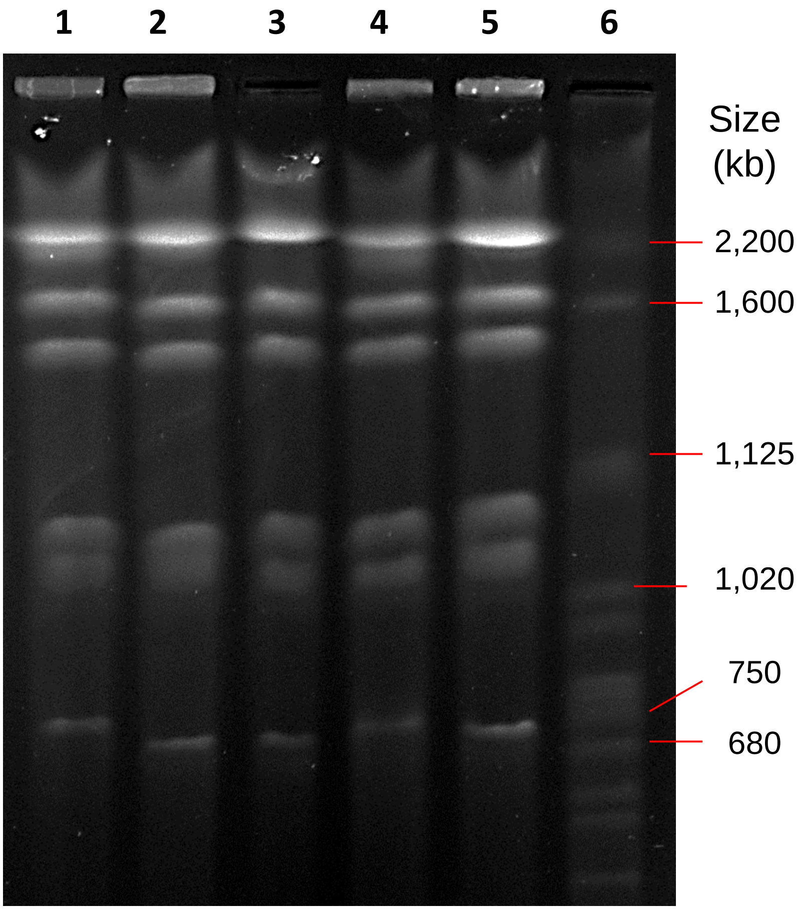

Supplementary Figure S7: Karyotypes of *Hanseniaspora* strains.
1: *H. pseudoguillermondii* CBS 8772^{T}, 2: *H. opuntiae* MUCL 49139^{T}, 3-5: *H. opuntiae* x *H. pseudoguillermondii* hybrids CLIB 3101, CCY46-1-3 and DBVPG 5828, respectively. 6: *S. cerevisiae* CLIB 112 (=YNN295) was used as molecular marker.
