# Supplementary material for: Investigation of Genetic Relationships Between Hanseniaspora Species Found in Grape Musts Revealed Interspecific Hybrids With Dynamic Genome Structures
Source: Front Microbiol. 2020 Jan 15;10:2960. doi: 10.3389/fmicb.2019.02960 (PMC6974558; doi:10.3389/fmicb.2019.02960)
Supplement: Supplementary file 8 [file Data_Sheet_8.PDF]

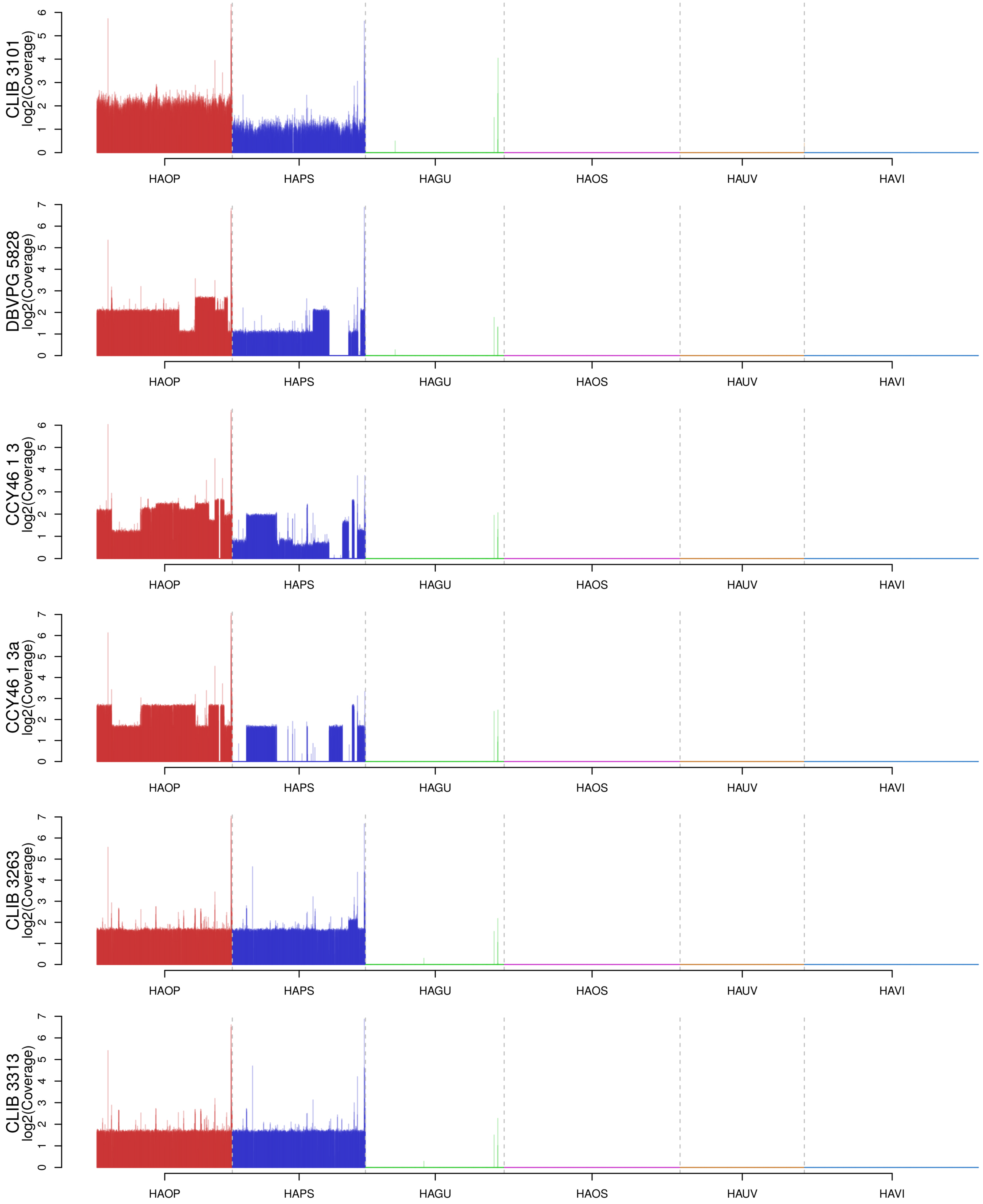

Supplementary Figure S8: Read coverage from the six hybrid strains along the genomes of *Hanseniaspora* strains. *H. opuntiae* (red) and *H. pseudoguilliermondii* (blue), *H. guilliermondii* (green), *H. osmophila* (pink), *H. uvarum* (brown), *H. vineae* (blue). Mean coverage values were computed with SppIDer tool, with a sliding-window of 1700 nucleotides (without overla**p).**
