# Supplementary material for: Investigation of Genetic Relationships Between Hanseniaspora Species Found in Grape Musts Revealed Interspecific Hybrids With Dynamic Genome Structures
Source: Front Microbiol. 2020 Jan 15;10:2960. doi: 10.3389/fmicb.2019.02960 (PMC6974558; doi:10.3389/fmicb.2019.02960)
Supplement: Supplementary file 9 [file Data_Sheet_9.PDF]

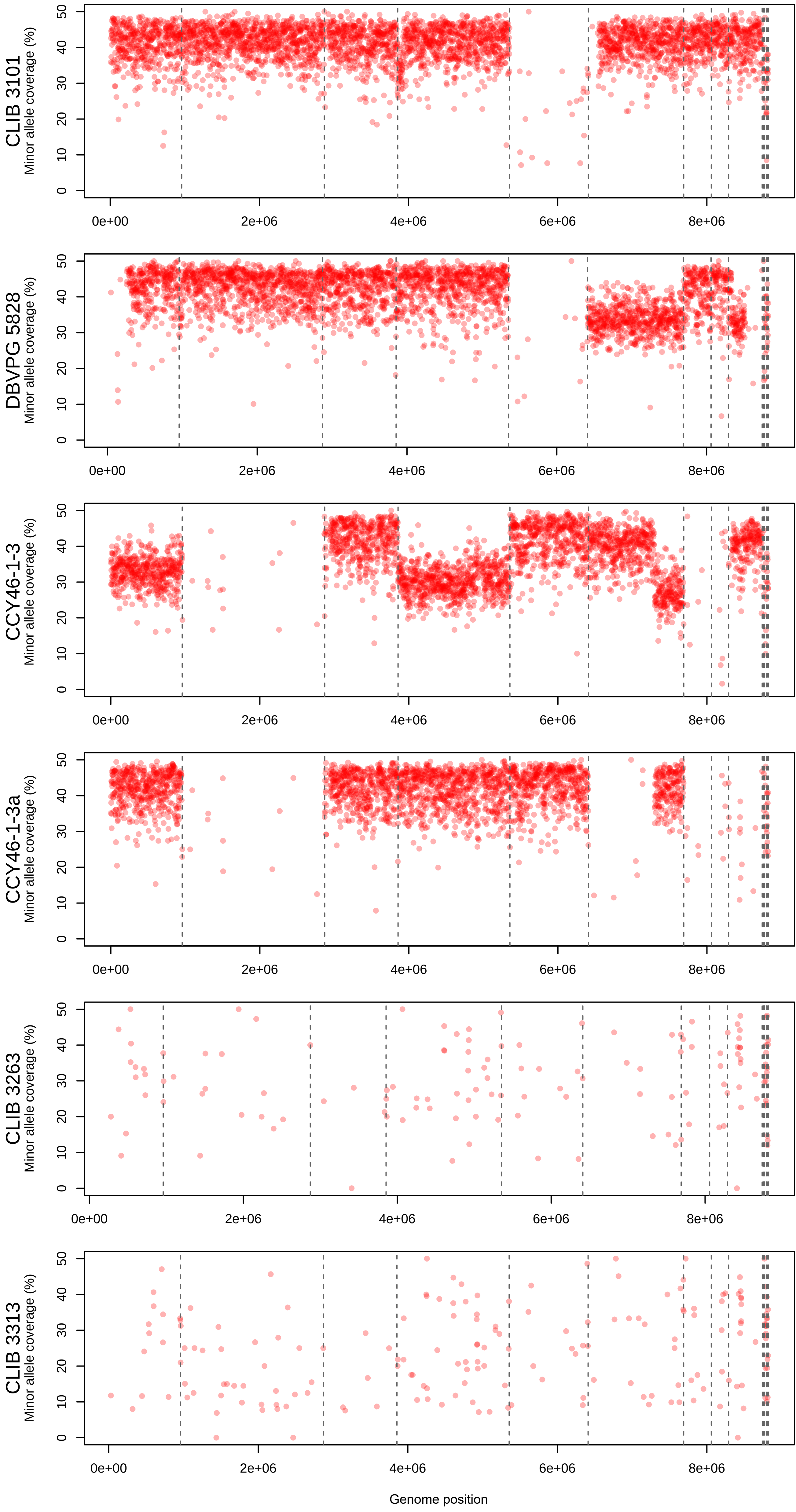

Supplementary Figure S9: Minor allele coverage (in % of the total coverage) for the 6 hybrid strains on the genome of *H. opuntiae*. Each dot corresponds to the mean minor allele coverage computed on 2-kb sliding windows (non-overlapping). Dashed grey lines indicate *H. opuntiae* scaffold boundaries.
