# Supplementary material for: Investigation of Genetic Relationships Between Hanseniaspora Species Found in Grape Musts Revealed Interspecific Hybrids With Dynamic Genome Structures
Source: Front Microbiol. 2020 Jan 15;10:2960. doi: 10.3389/fmicb.2019.02960 (PMC6974558; doi:10.3389/fmicb.2019.02960)
Supplement: Supplementary file 10 [file Data_Sheet_10.PDF]

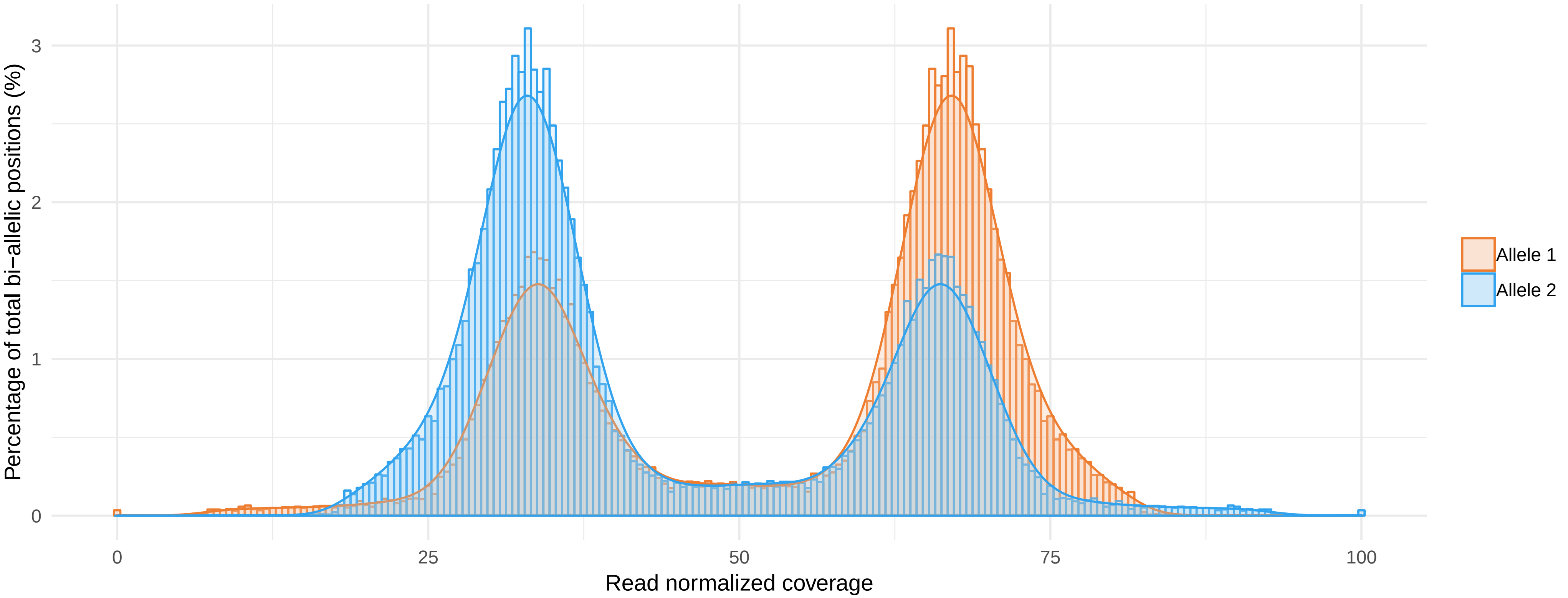

Supplementary Figure S10: Distributions of read normalized coverage at bi−allelic positions in the genome of *H. uvarum* strain S382-CB. Results enclose read coverage values of Reference/Alternative1 positions (55,355) as well as Alternative1/Alternative2 positions (160).
