# Supplementary material for: Investigation of Genetic Relationships Between Hanseniaspora Species Found in Grape Musts Revealed Interspecific Hybrids With Dynamic Genome Structures
Source: Front Microbiol. 2020 Jan 15;10:2960. doi: 10.3389/fmicb.2019.02960 (PMC6974558; doi:10.3389/fmicb.2019.02960)
Supplement: Supplementary file 3 [file Data_Sheet_3.PDF]

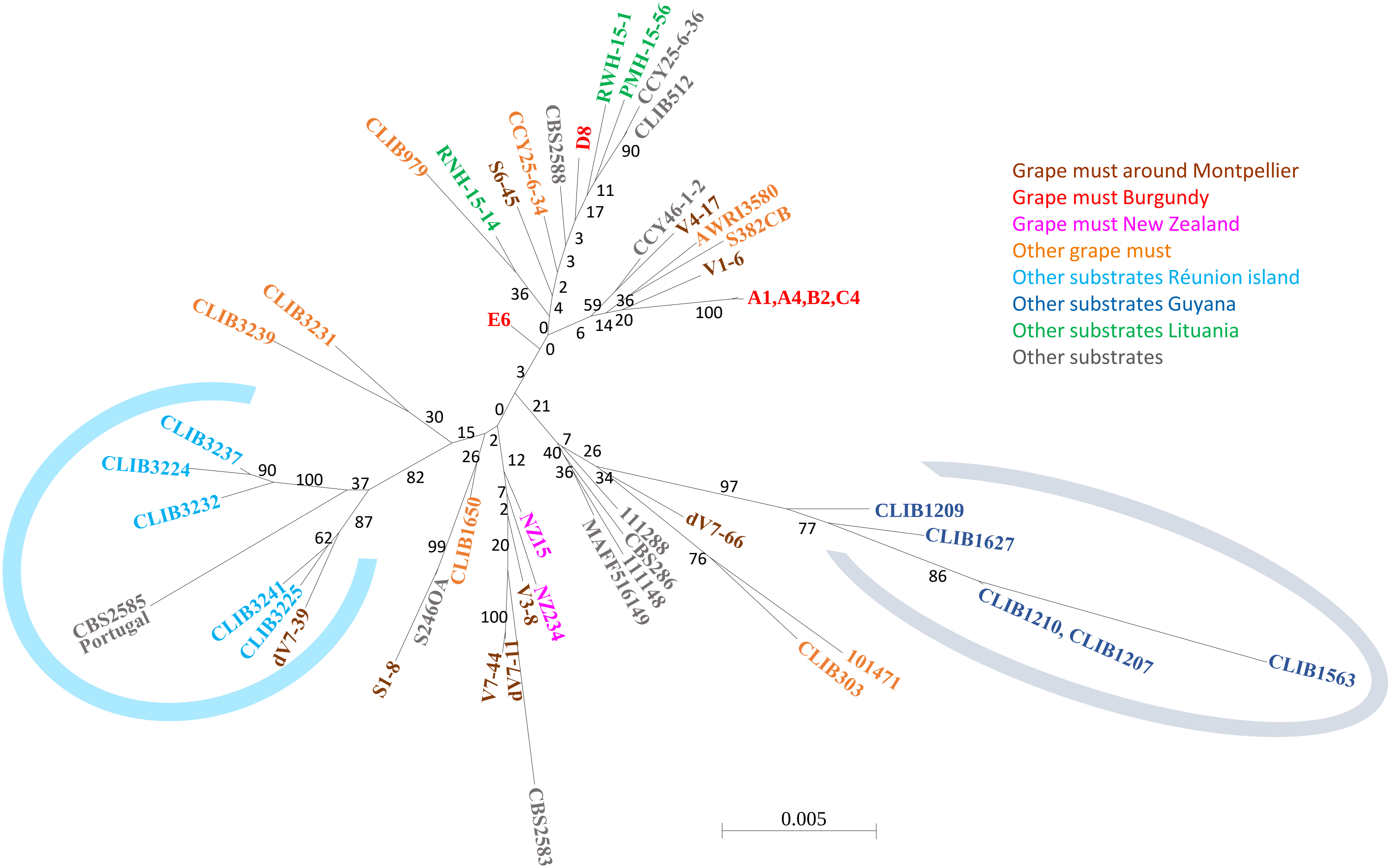

Supplementary figure S3: Phylogenetic tree of *H. uvarum* based on the concatenation of the five MLST markers. The unrooted tree was reconstructed with PhyML from the alignment of 3430 residues. Strain names are coloured according to their geographical and/or substrate origin: Brown for grape musts in Montpellier region (France), red for grape musts in Burgundy (France), pink for grape musts in New Zealand, orange for grape musts from other countries, turquoise for other substrate in La Réunion Island, blue for other substrates in Guyana, green for other substrates in Lituania, and grey for other substrates in other countries. The well-supported branch of strains isolated from Guyana is circled in blue, and the branch of strains enriched in strains from La Réunion island in turquoise.
